# Unveiling structural determinants of peptide recognition by public and private SARS-CoV-2-specific T-cell receptors

**DOI:** 10.1038/s41467-026-77827-z

**Published:** 2026-09-22

**Authors:** Caner Akıl, Yanchun Peng, Julia McCarthy, Yanan Zhu, Nathan Hardenbrook, Yunkai Yang, Danning Dong, Elie Antoun, Xuan Yao, Guihai Liu, Ji-li Chen, Esra Balıkçı, Raul Cioaca, Alexander J. Mentzer, Julian C. Knight, Ricardo A. Fernandes, Tao Dong, Peijun Zhang

**Affiliations:** 1 Chinese Academy of Medical Sciences Oxford Institute, Nuffield Department of Medicine, University of Oxford, Oxford, UK; 2 Division of Structural Biology, Nuffield Department of Medicine, University of Oxford, Oxford, UK; 3 MRC Translational Immune Discovery Unit, MRC Weatherall Institute of Molecular Medicine, Radcliffe Department of Medicine, University of Oxford, Oxford, UK; 4 Institute for Advanced Study in Physics, Zhejiang University, Hangzhou, Zhejiang China; 5 Wellcome Centre for Human Genetics, Nuffield Department of Medicine, University of Oxford, Oxford, UK; 6 Diamond Light Source, Harwell Science and Innovation Campus, Didcot, UK

**Keywords:** Viral infection, Cryoelectron microscopy, MHC class I, T-cell receptor, Immunological memory

## Abstract

Public T cell receptors (TCRs) recurrently emerge across individuals in response to common pathogens, yet the structural and biophysical basis distinguishing public from private clonotypes remains incompletely defined. Here, we combine epitope mapping, single-cell TCR sequencing, and single-particle cryo-electron microscopy to dissect CD8^+^ T cell responses to the immunodominant SARS-CoV-2 ORF3a_(207-215)_ epitope presented by HLA-A*01:01. Among responding clonotypes, we identify a public TCR (TCR_pub_), shared across donors, and a private TCR (TCR_priv_) that uses nearly identical β chains but distinct α chains. Despite comparable micromolar affinities and functional avidities, cryo-EM structures at ~3 Å resolution reveal that the two receptors engage the peptide-MHC complex through distinct CDR-loop configurations and contact footprints. TCR_pub_ establishes contacts with the peptide and the MHC α2-helix primarily through the CDR2β and CDR3β loops, whereas TCR_priv_ distributes contacts across both MHC α-helices and engages the peptide via multiple CDR loops. In agreement with these observations, alanine-scanning mutagenesis reveals that TCR_pub_ shows greater sensitivity to peptide substitutions, consistent with its peptide-focused binding mode. These findings demonstrate how alternative α-chain pairing reconfigures the antigen-binding interface within a shared β-chain.

## Abstract (web summary)

Public TCR receptors are shared across individuals, but private TCRs are more restricted. Here the authors look at the biophysical differences between a public and a private TCR that recognise an HLA-A*01:01 restricted SARS-CoV-2 ORF3a epitope and show divergent alpha chains, differing engagement of CDR-loop configurations and contact footprints.

## Introduction

T cells are central to adaptive immunity, detecting infected or malignant cells through TCRs that engage peptide-MHC (pMHC) complexes on target cells^1–6^. Somatic recombination of variable (V), diversity (D), and joining (J) gene segments generates vast receptor diversity, which is refined by thymic selection to yield a peripheral repertoire of ~10^8^ clonotypes capable of broad pathogen recognition while maintaining self-tolerance^7–11^. Despite this immense diversity, TCRs with identical or highly similar sequences, termed public TCRs, often recur across unrelated individuals responding to the same antigen. Such convergence likely reflects recombination biases and selection pressures that favour αβ pairings with optimal structural complementarity to specific pMHCs, leading to advantageous functional outcomes. Strikingly, public TCR responses are frequently observed against immunodominant epitopes from common viral infections, including SARS-CoV-2^12–18^. Previous studies, including our own, have shown that public TCRs are associated with high-frequency, pre-existing precursors and may contribute to rapid and broad early immune responses^14,19–21^. Public clonotypes have also been implicated in autoimmune diseases, but remain notably rare in cancer, despite extensive repertoire profiling efforts^22–24^. In contrast, private TCRs, which are unique to individuals, dominate most immune responses. However, the structural and functional distinctions between public and private clonotypes, and how these differences influence recognition of identical peptide-MHC ligands, remain poorly defined^25^.

Although thousands of TCR sequences with known antigen specificity have been catalogued, structural coverage remains limited and biased^26^. Nevertheless, key principles of TCR/pMHC engagement have emerged: hypervariable CDR3 loops primarily contact the bound peptide, whereas germline-encoded CDR1 and CDR2 loops interact mainly with the MHC helices^27–33^. Most TCR/pMHC complexes have been determined by X-ray crystallography, which has formed the foundation of our current understanding of TCR recognition.

The available structural dataset reflects experimental, biological, and demographic biases, including unequal TRAV/TRBV gene usage and limited HLA diversity^26^, with a notable enrichment for European ancestry. Together with constraints associated with crystallization, these factors contribute to the underrepresentation of certain TRAV-TRBV pairings^24,26,33^. Single-particle cryo-electron microscopy (cryo-EM) has strong potential as a complementary approach for studying macromolecular complexes in solution, despite challenges related to small size, preferred orientation, and conformational heterogeneity. Recent advances have extended its applicability to smaller assemblies, including TCR/pMHC complexes.

To explore how closely related clonotypes can adopt distinct antigen-recognition modes, we examined two CD8^+^ T cell receptors from convalescent SARS-CoV-2 patients that recognise the same ORF3a_(207-215)_ epitope (FTSDYYQLY), presented by the common HLA-A*01:01 allomorph. Recognition of this epitope elicits strong and frequent CD8^+^ T cell responses in convalescent individuals, with a high degree of clonotype sharing across donors. Within this response, we identified two dominant clonotypes, one public (TCR_pub_; TRAV3/TRBV5-1) and one private (TCR_priv_; TRAV25/TRBV5-1), that recognise the same pMHC ligand yet differ only in their α-chain usage and four CDR3β residues. This natural pair provides an ideal system to dissect how α-chain pairing and subtle CDR3 differences shape antigen recognition within a shared β-chain scaffold.

Here, we combine epitope mapping, single-cell TCR sequencing, affinity measurements, and cryo-EM to determine the structures of TCR_pub_ and TCR_priv_ bound to ORF3a_(207-215)_/HLA-A*01:01. Despite comparable micromolar affinities and similar overall docking architectures, the two receptors engage the peptide-MHC surface through divergent CDR-loop configurations. Together, our findings show how alternative α-chain pairings reconfigure the antigen-binding interface and shape antigen recognition within a shared β-chain framework, while also demonstrating that cryo-EM provides a powerful complementary approach for the structural determination of micromolar-affinity TCR/pMHC complexes.

## Results

### Dominant SARS-CoV-2 ORF3a_(207-215)_-HLA-A*01:01-specific T cells show biased TCR usage and include a public TCR clonotype

**Fig. 1:**
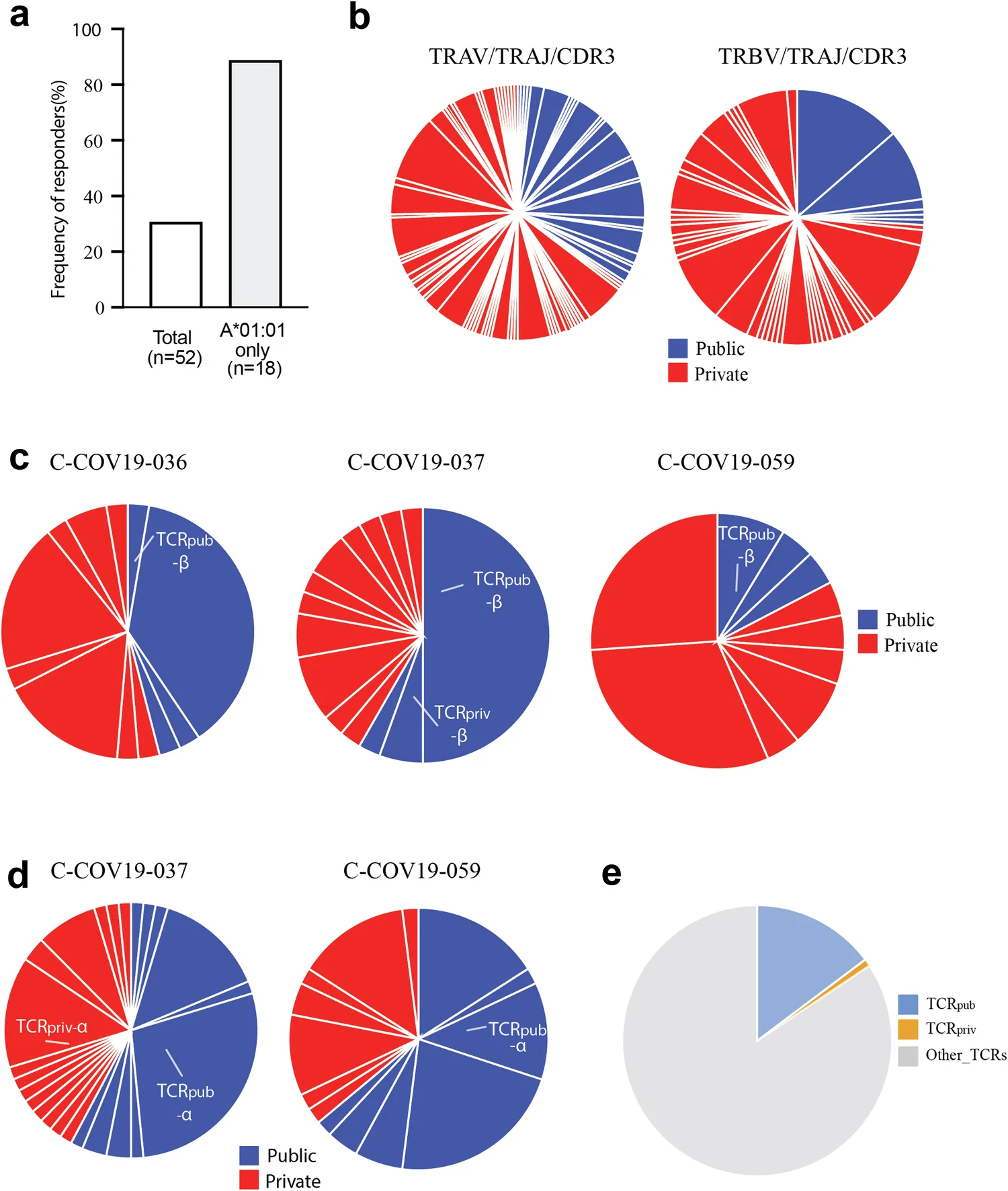
Identification of a public TCR specific to ORF3a_(207-215)_/HLA-A*01:01. **a** Frequency of responders to the HLA-A01:01-restricted ORF3a_(207-215)_ epitope (FTSDYYQLY). **b** Frequency of public T cell receptors by TRAV/TRAJ/CDR3A (left) and TRBV/TRBJ/CDR3B (right) usage in ORF3a_(207-215)_/HLA-A01:01-specific CD8⁺ T cells. **c** Frequencies of TCR_pub_ β chain and TCR_priv_ β chain in three individuals. **d** Frequencies of TCR_priv_ and TCR_pub_ α chains in four individuals. **e** Frequencies of paired TCR_priv_ and TCR_pub_ in ORF3a_(207-215)_/HLA-A*01:01-specific cells across four individuals. TCR_priv_ = 1, TCR_pub_ = 17, other TCRs = 94. Source data are provided as a Source Data file.

Previous studies conducted by us and others have identified a dominant CD8^+^ epitope ORF3a_(207-215)_ (FTSDYYQLY) derived from the SARS-CoV-2 ORF3a protein, restricted by HLA-A*01:01^34–37^. In our cohort, 52 individuals who recovered from COVID-19 were HLA-typed, and 18 (34.6%) were HLA-A*01:01 positive. An ex vivo interferon (IFN)-γ ELISpot assay showed that 88.9% (16/18) of these individuals responded to this epitope, representing 30.8% (20/52) of the overall cohort (Fig. 1a and Supplementary Table 1**)**. To characterize this dominant T cell response, ORF3a_(207-215)_-A*01:01-specific T cells from four donors were sorted at the single-cell level by flow cytometry using peptide-MHC Pentamer for Smartseq2 sequencing (Supplementary Fig. 1a, 1b). Analysis of their TCR usage revealed a high frequency of shared TCR chains detected in more than one individual (Supplementary Data 1). As shown in Fig. 1b, c, 26 out of 85 TRA (30.6%) chains and 6 out of 46 β chains (13%) were shared across multiple individuals. When considering both αβ chains, only one of these corresponded to a public TCR clonotype, defined here as a shared identical paired αβ TCR sequence detected across individuals.

Among these ORF3a_(207-215)_-A*01:01-specific TCRs, we chose two for downstream characterisation based on their ab usage and frequency across individuals (Supplementary Table 2): both TCRs utilise the same CDR1b and CDR2b sequences (TRBV5-1/TRBJ1-1) with slightly different CDR3 variable regions and pair with distinct α chains. Sequence alignments confirmed that, as expected from their shared TRBV gene usage, the TCR β-chains are highly conserved between these clonotypes, with differences limited to the four amino acids in the CDR3β loop. In contrast, the TCR α-chains exhibit greater overall sequence diversity (Supplementary Fig. 2a, b). One of these TCRs was classified as a public TCR (TCR_pub_) because the paired αβ clonotype was detected in two individuals. TCR_pub_ contains a shared β chain that was detected in three individuals: C-COV19-036 (2.70%), C-COV19-037 (50%), and C-COV19-059 (8.7%). By contrast, TCR_priv_ contains a β chain detected only in donor C-COV19-037 (5.56%) (Fig. 1c). At the α-chain level, TCR_priv_ uses a unique TRAV25/TRAJ57 chain detected only in C-COV19-037, whereas TCR_pub_ uses a shared TRAV3/TRAJ6 α chain detected in both C-COV19-037 and C-COV19-059 (Fig. 1d). Within donor C-COV19-037, TCR_pub_ was detected at a higher frequency (17.5%) than TCRpriv (1%) (Fig. 1e).

**Fig. 2:**
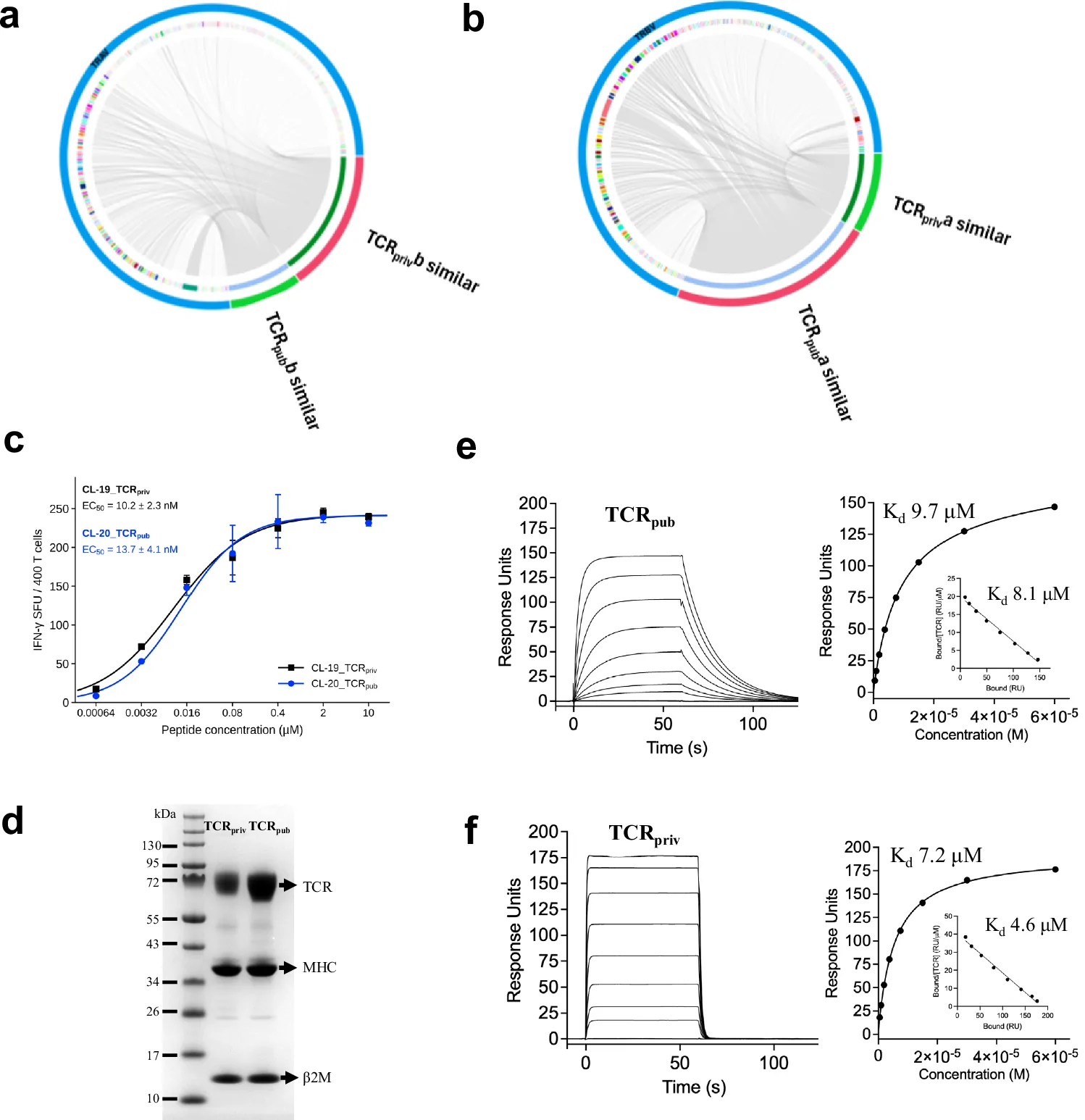
Biochemical characterization of TCR/pMHC complexes and TCR repertoire similarity in COVID OTS dataset. **a**, **b** CDR3 sequence similarity and α-β pairing diversity among OTS COVID TCRs. **a** Circos plot showing TRAV gene usage and α-chain pairing of OTS COVID TCRs that share similar β CDR3 sequences with TCR_priv_ and TCR_pub_ (Levenshtein similarity > 0.8). TCRs with CDR3β similar to TCR_priv_ (148 sequences) and TCR_pub_ (92 sequences) are indicated as TCR_priv_b_similar (pink) and TCR_pub_b_similar (green), respectively. Their paired α chains are connected by grey links, illustrating highly diverse α chain usage (244 and 146 unique α CDR3s, respectively). **b** Circos plot showing TRBV gene usage and β-chain pairing of OTS COVID TCRs that share similar α CDR3 sequences with TCR_priv_ and TCR_pub_. TCRs with α CDR3 similar to TCR_priv_ (94 sequences) and TCR_pub_ (45 sequences) are shown as TCR_priv_a_similar (pink) and TCR_pub_a_similar (green). Grey links connect these α-similar TCRs to their paired β chains (196 and 83 unique CDR3βs, respectively), revealing a more conserved α-β pairing pattern in TCR_pub_-like repertoires. **c** IFN-γ ELISpot peptide titration of ORF3a(207–215)-HLA-A*01:01-specific T cell clones CL-19 (TCRpriv, black squares) and CL-20 (TCRpub, blue circles), co-cultured with peptide-pulsed target cells. Symbols are the mean of the two independent experiments, and error bars are the SEM. Each biological replicate is the average of *n* = 2 technical replicates. The curve is drawn from the mean of the parameters fitted separately to each experiment. **d** SDS-PAGE gel (4–12% Bis-Tris gradient gel, run with MES buffer) showing purified TCR_pub_/pMHC and TCR_priv_/pMHC complexes under reducing conditions. The gel was used as a quality control assessment of the purified recombinant protein complexes prior to downstream biochemical and structural analyses. Left: SPR sensorgrams showing binding of eight concentrations of TCR_pub_ (**e**) and TCR_priv_ (**f**) (starting at 60 μM with 2-fold serial dilutions) to 220-250 RU of immobilized ORF3a₍₂₀₇₋₂₁₅₎–HLA-A*01:01 complexes at 37 °C. Responses from a reference flow cell containing an irrelevant pMHC were subtracted from the total signals. Right: Nonlinear curve fitting of the untransformed data using a 1:1 Langmuir binding model yielded a Kd = 7.2 μM and Bmax = 176 RU for TCR_pub_ (**e**), and Kd = 9.7 μM and Bmax = 147 RU for TCR_priv_, respectively. A linear Scatchard plot (inset) produced a similar Kd = 4.6 μM (**e**) and Kd = 8.1 μM (**f**). Data are representative of three independent experiments. Source data are provided as a Source Data file.

We also analysed the COVID-19 OTS dataset^24^, and this analysis revealed that 148 and 92 TCRs shared CDR3β sequences similar to TCR_priv_ and TCR_pub_, respectively (Levenshtein similarity score > 0.8). The β chains in these TCRs were paired with 244 and 146 unique α chains, respectively, indicating a greater α-chain diversity among TCR_priv_-like TCRs. Conversely, 94 and 45 TCRs exhibited CDR3α similarity to TCR_priv_ and TCR_pub_, pairing with 196 and 83 unique β chains, respectively. The maximum cross-chain similarity scores were 0.733 (α) and 0.692 (β) for TCR_priv_, and 0.714 (α) and 0.769 (β) for TCR_pub_, suggesting that while none of the pairings reached high absolute similarity, distinct diversity patterns emerged between the two receptors. Overall, TCR_priv_-like repertoires displayed broader combinatorial diversity with highly variable α-β pairings. In contrast, TCR_pub_-like repertoires were characterized by more recurrent and conserved α-β combinations, consistent with their classification as private and public TCRs, respectively (Fig. 2a, b).

### TCR_pub_ and TCR_priv_ recognise ORF3a_(207-215)_-HLA-A*01:01 with micromolar affinity and comparable functional avidity

To assess the functional sensitivity of TCR_pub_ - and TCR_priv_-expressing T cell clones, we evaluated IFN-γ responses using an ELISpot assay. ORF3a_(207-215)_**-**A*01:01-specific T cell clones were isolated by single-cell peptide-MHC multimer sorting, in vitro expansion and validated by multimer staining. Clones with >95% purity were used for experiments (Supplementary Fig. 1b and 1c). Clone 19 (TCR_priv_) and Clone 20 (TCR_pub_) exhibited similar functional avidities, with EC_50_ values of 10.2 nM and 13.7 nM, respectively (Fig. 2c and Supplementary Fig. 1d). To dissect how TCR_pub_ and TCR_priv_ clonotypes engage ORF3a_(207-215)_-A*01:01 (hereafter referred to as the peptide-MHC complex, pMHC), we generated soluble TCR heterodimers by removing the transmembrane regions and co-expressing the ectodomains of α- and β-chains in mammalian cells (Supplementary Fig. 3a). These soluble receptors enabled both biophysical and structural interrogation of ORF3a-specific TCR/pMHC interactions (Fig. 2d and Supplementary Fig. 3b–d). Binding affinities of TCR_pub_ and TCR_priv_ to the same cognate pMHC complex were quantified by surface plasmon resonance (SPR), yielding dissociation constants in the micromolar range (7.2–9.7 µM) (Fig. 2e, f), similar to the affinities reported for other antiviral TCRs^38^. To further characterize the biochemical properties of the soluble, recombinant TCRs, mass photometry analysis of TCR_pub_/pMHC and TCR_priv_/pMHC complexes revealed that both complexes are predominantly monomeric, with a small fraction (~5%) of TCR_pub_/pMHC complexes exhibiting a dimer configuration (Supplementary Fig. 3e, f).

### Cryo-EM structures of TCR_pub_ and TCR_priv_ in complex with ORF3a_(207-215)_-HLA-A*01:01

**Fig. 3:**
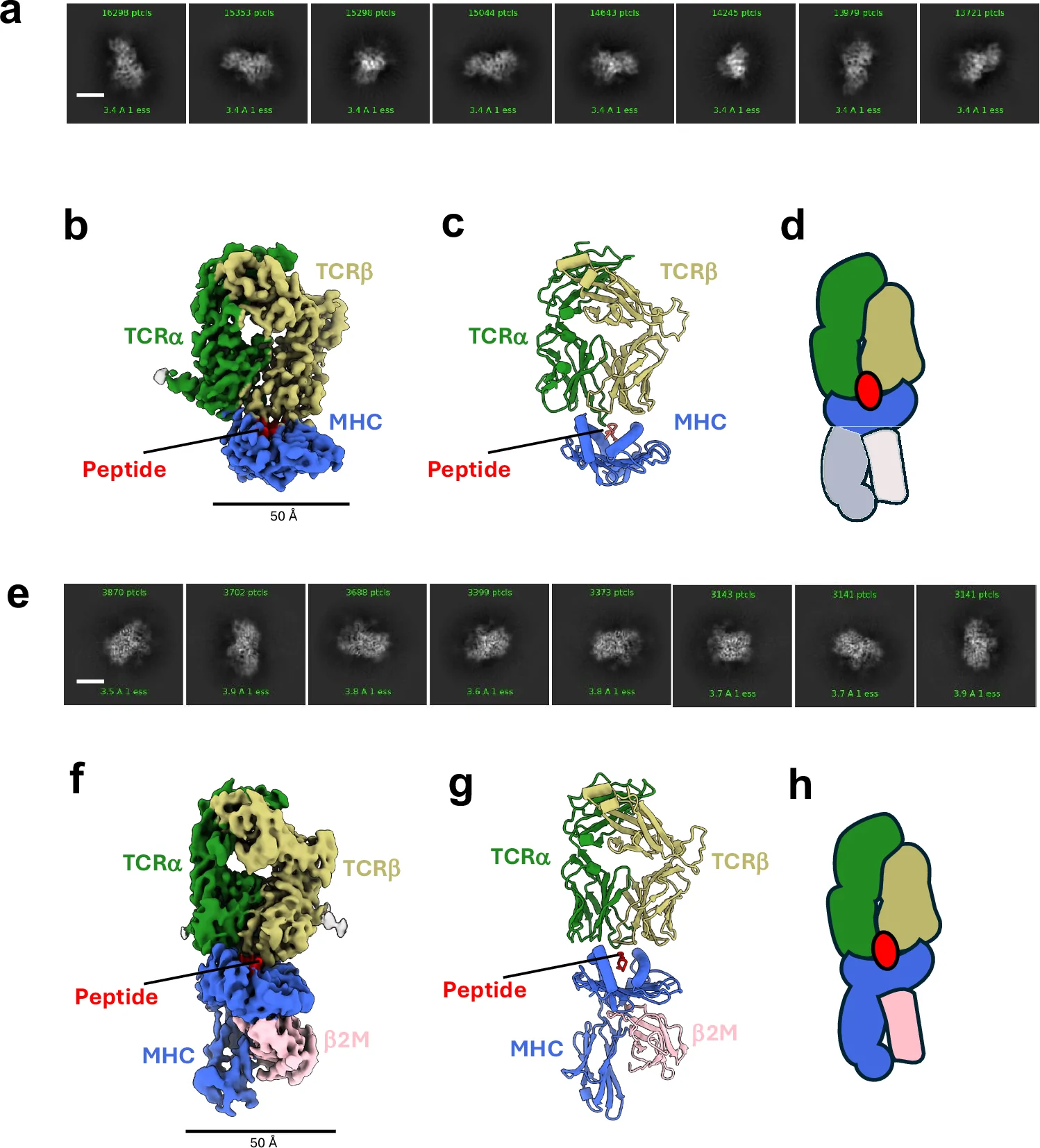
Cryo-EM structures of TCR_pub_/pMHC and TCR_priv_/pMHC complexes. **a** Representative 2D class averages of TCR_pub_/pMHC. **b** Cryo-EM density map of TCR_pub_/pMHC with subunits shown in indicated colours. **c** Ribbon diagram of the TCR_pub_/pMHC structural model. **d** Cartoon schematic of TCR_pub_/pMHC. The TCRα chain is depicted in green, the TCRβ chain in dark khaki, the peptide ligand in red, and the MHC molecule in blue. The MHC α3 domain (dark grey) and β2-microglobulin (light grey) were not well resolved. **e** Representative 2D class averages of TCR_priv_/pMHC. **f** Cryo-EM density map of TCR_priv_/pMHC with subunits shown in indicated colours. **g** Ribbon diagram of the TCR_priv_/pMHC structural model. Cryo-EM density maps are displayed at contour levels of 0.136 for the TCR_pub_/pMHC focused map and 0.0008 for the TCR_priv_/pMHC map. **h** Simplified cartoon schematic of TCR_priv_/pMHC. Scale bars in a and e (white), and in b and f (black) indicate 50 Å.

**Fig. 4:**
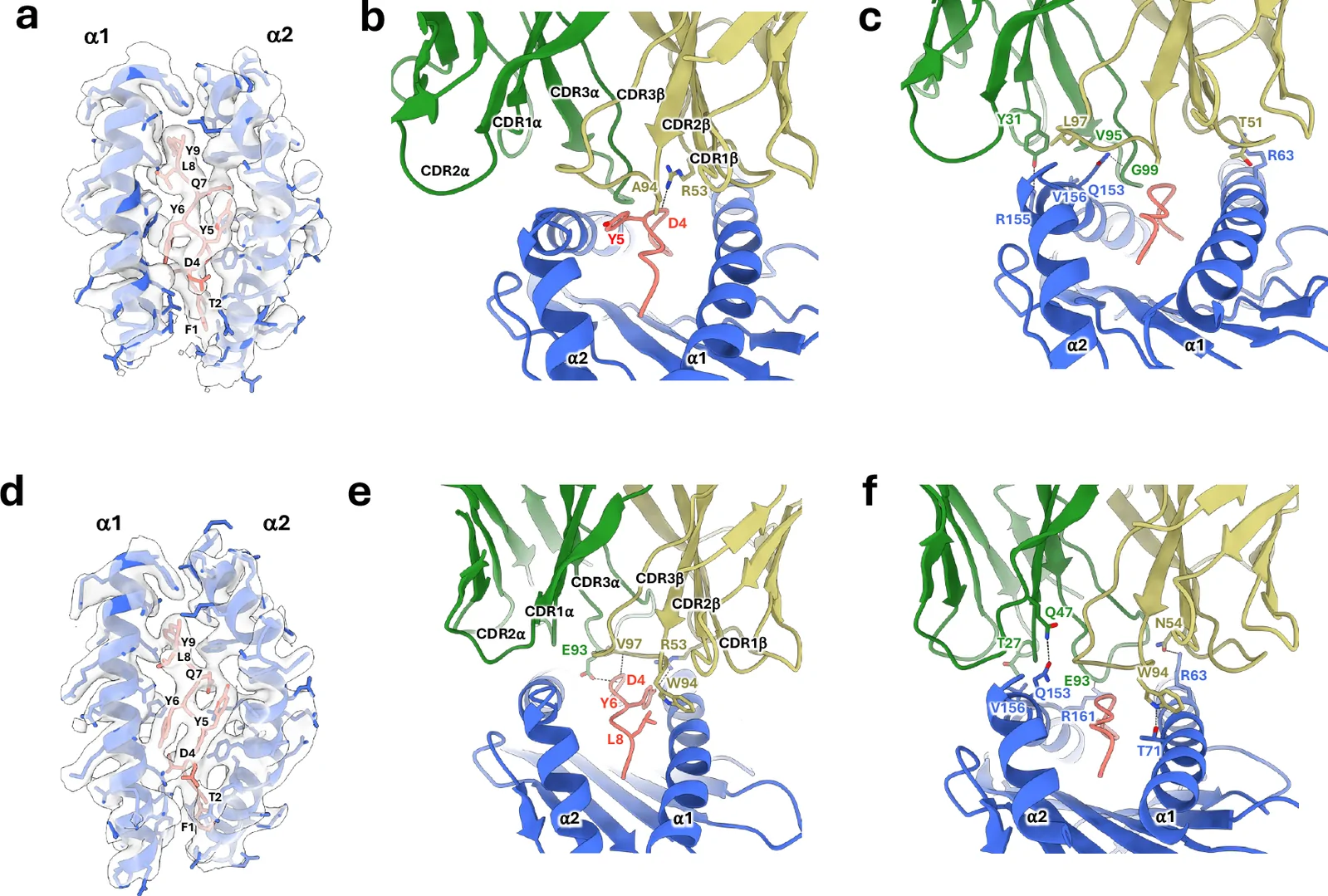
TCR recognition of the ORF3a_(207-215)_ peptide and MHC molecule. Structural details of the TCR_pub_/pMHC complex with density and model superimposed, shown at the ORF3a_(207-215)_ peptide (FTSDYYQLY) and MHC interface (**a**), the interface between TCR_pub_ CDR loops and the peptide with interacting residues labelled (**b**), and the interface between TCR_pub_ CDR loops and MHC with interacting residues labelled (**c**). Structural details of the TCR_priv_/pMHC complex with density and model superimposed, shown at the peptide and MHC interface (**d**), the interface between TCR_priv_ CDR loops and the peptide with interacting residues labelled (**e**), and the interface between TCR_priv_ CDR loops and MHC with interacting residues labelled (**f**). The TCRα chain is shown in green, the TCRβ chain in dark khaki, the peptide ligand in red, and the MHC molecule in blue. The TCRα chain CDR loops (CDR1α, CDR2α, and CDR3α) and TCRβ chain CDR loops (CDR1β, CDR2β, and CDR3β) are marked.

Next, we performed single-particle cryo-EM analysis of TCR_pub_/pMHC, TCR_priv_/pMHC in complex with the ORF3a_(207-215)_ epitope. To address the challenges associated with structural determination of these natural TCR/pMHC complexes, including their small size (~93 kDa), inherent flexibility, micromolar-affinity, preferred orientation, and particle disassembly at the air-water interface, we collected a large dataset for each complex (4-6 million particles) and additionally recorded images at a 30° tilt angle. To minimize particle heterogeneity and orientation bias, we further carried out iterative rounds of particle picking, followed by stringent two-dimensional (2D) and three-dimensional (3D) classifications (Supplementary Fig. 4 and 5, Supplementary Table 3). Final reconstructions produced cryo-EM maps of TCR_pub_/pMHC and TCR_priv_/pMHC complexes, with global resolutions of 3.17 Å and 3 Å, respectively (Supplementary Fig. 4, 5). The resulting cryo-EM maps of TCR_pub_/pMHC and TCR_priv_/pMHC exhibited well-resolved features for the TCR α and β chains, the ORF3a_(207-215)_ peptide, and the α1/α2 helices of the MHC (Fig. 3). In the TCR_pub_/pMHC complex, density for the membrane-proximal MHC α3 domain and β2-microglobulin was less well resolved compared to the peptide-binding platform, suggesting regional flexibility or conformational heterogeneity in this portion of the complex. In contrast, these regions were well resolved in the TCR_priv_/pMHC reconstruction. To address this, focused refinement using masks encompassing the TCR-peptide-MHC α1/α2 interface and the MHC β-sheet platform was performed, yielding a map of sufficient quality to allow confident model building of the TCR, peptide, and MHC α1/α2 domains. This enabled detailed analysis of the antigen recognition interface in the TCR_pub_ complex. In both TCR_pub_/pMHC and TCR_priv_/pMHC complexes, the ORF3a_(207-215)_ peptide is buried in the cleft formed by the MHC α1 and α2 helices, as expected. The peptide is mainly contacted by the CDR3α and CDR3β loops (Fig. 4b, e), while the MHC is primarily engaged by CDR1 and CDR2 loops of both TCRα and TCRβ chains (Fig. 4c, f).

The local resolution at the peptide-binding cleft in both the TCR_pub_/pMHC and TCR_priv_/pMHC complexes was sufficiently high to allow confident modelling of side chains (Fig. 4 and Supplementary Fig. 6), enabling detailed analysis of the molecular interactions of these wild-type TCR/pMHC complexes that underpin T cell recognition. The conformations of the MHC, the ORF3a_(207-215)_ peptide, and their interfaces were nearly identical in both complexes (Fig. 4a, d). The N-terminus of the peptide forms hydrogen bonds with Tyr residues on the MHC, while Asp4, Tyr5, and Tyr6 engage the MHC helices and contribute to stabilizing the peptide-MHC interaction (Fig. 4a, d and Supplementary Fig. 6).

### Antigen recognition interfaces of TCR_pub_ and TCR_priv_

Detailed interface analysis revealed that both TCR_pub_ and TCR_priv_ engage the ORF3a_(207-215)_ peptide through distinct combinations of peptide- and MHC-mediated contacts. In both complexes, Arg53 of the CDR2β loop serves as a central anchoring residue at the peptide interface, but its mode of engagement differs. In TCR_pub_, the backbone of peptide residue Asp4 forms a hydrogen bond with Arg53, and Ala94 of the CDR3β loop (within the AGDL motif) makes a moderate hydrophobic contact with Tyr5 of the peptide (Fig. 4b). Together, these interactions concentrate peptide recognition through a limited set of CDR3β contacts. In contrast, the TCR_priv_ CDR loops engage the peptide more extensively: Tyr6 forms a hydrogen bond with Arg53 of CDR2β, and the backbone of Asp4 is positioned to form an additional salt bridge with Arg53, stabilising the central interface (Fig. 4e). Further backbone-backbone hydrogen bonds are observed between Asp4 and Val97 (CDR3β), and Glu93 (CDR3α) (Fig. 4e). Furthermore, Leu8 of the peptide engages Trp94 of the WTGV CDR3β motif through hydrophobic interactions. The tryptophan, replaced by alanine in TCR_pub_, provides the broader peptide contact footprint of TCR_priv_.

The two receptors also differ in how they distribute contacts across the MHC α-helices. TCR_pub_ concentrates its MHC interactions on the α2 helix: Val156 forms a hydrophobic contact with Val95 (CDR3α), while Gln153 and Arg155 form hydrogen bonds with Gly99 (CDR3α) and Tyr31 (CDR1α), respectively. A hydrophobic interaction between Thr51 (CDR2β) and Arg63 of the α1 helix provides the sole α1-helix contact (Fig. 4c). In contrast, TCR_priv_ engages both α1 and α2 helices more evenly through multiple hydrogen bonds: Gln47 (CDR2α) with Gln153 (α2); Asn54 (CDR2β) with Arg63 (α1) via a salt bridge; the backbone of Glu93 (CDR3α) with Arg161 (α2); Trp94 (CDR3β) with Thr71 (α2); and Thr27 (CDR1α) with Val156 through a hydrophobic interaction (Fig. 4f). Collectively, these contacts reveal that TCR_pub_ adopts a peptide-focused, α2-centric recognition mode, whereas TCR_priv_ achieves a more balanced interface distributed across both the peptide and both MHC helices.

**Fig. 5:**
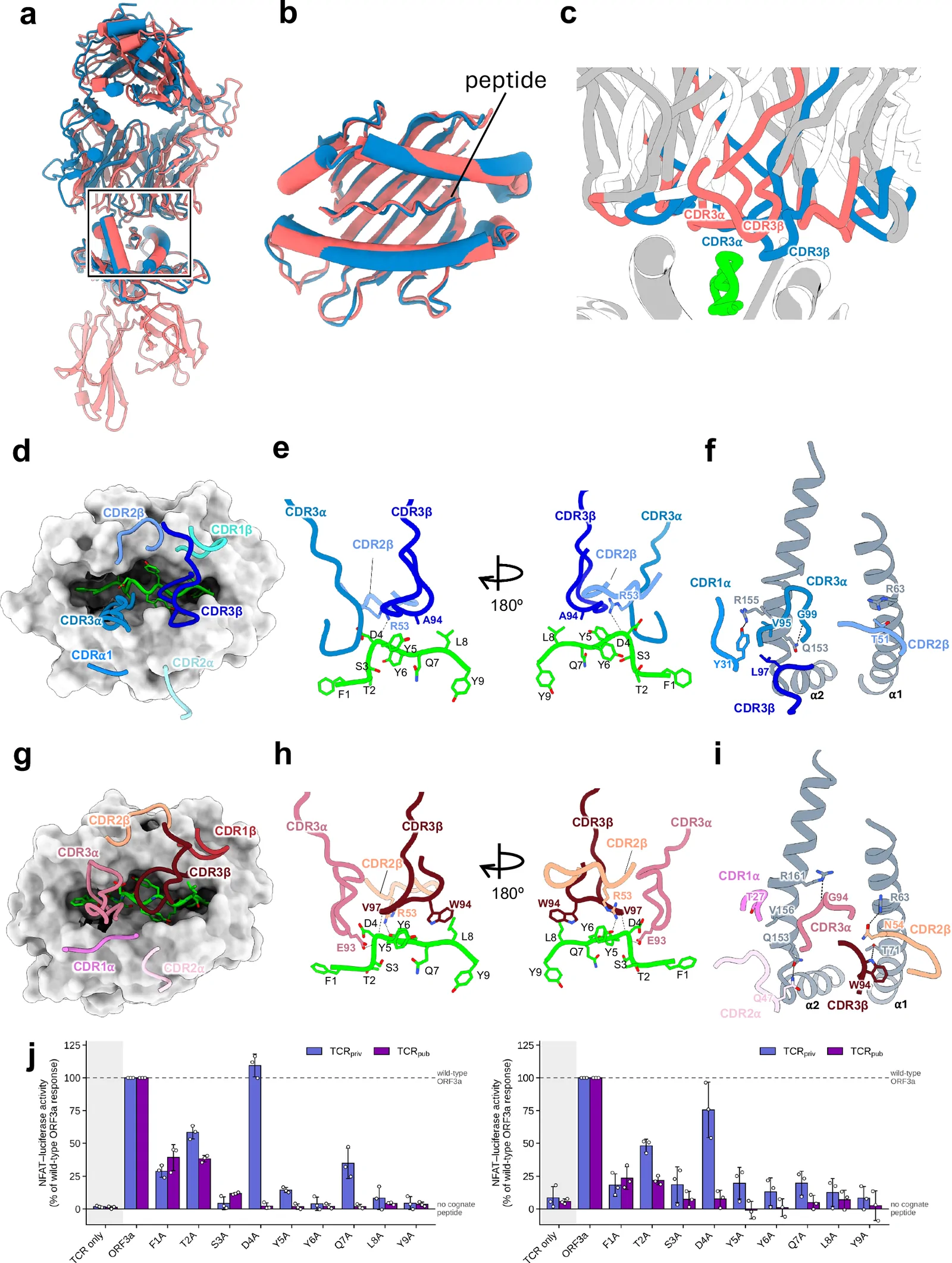
Comparative analysis of TCR_pub_ and TCR_priv_ engaging peptide antigen and MHC. **a** Superimposition of TCR_pub_/pMHC (blue) and TCR_priv_/pMHC (salmon) and complexes aligned on the pMHC. **b** Zoom-in on the aligned pMHC region. (**c**) Zoom-in on the CDR loops from TCR_pub_/ (blue) and TCR_priv_ (salmon). The peptide is shown in green. **d** Footprint of TCR_pub_/ CDR loops (blue tones) on the pMHC complex (grey surface). Interaction between the TCR_pub_ CDR loops (blue tones) and the peptide (green, **e**) and the MHC (grey, **f**). Hydrogen bonds are shown with dashed lines. **g** Footprint of TCR_priv_ CDR loops (red tones) on the pMHC complex (grey surface). Interaction between TCR_priv_ CDR loops and the peptide (green, **h**) and the MHC molecule (grey, **i**). (**j**) NFAT-luminescence-based readout of T cell activation for the indicated peptides. ORF3a denotes the wild-type peptide. All subsequent peptides correspond to single alanine substitution variants across the ORF3a sequence, as indicated. T2 cells expressing the HLA-A*01:01 (T2-A1) were pulsed with 1 µM or 10 µM peptide and co-cultured with Jurkat CD8 + NFAT-luciferase reporter cells expressing TCRpriv or TCRpub. Activation is expressed as a percentage of the wild-type ORF3a response after subtraction of the no-peptide (TCR + T2-A1) background, normalised within each biological replicate and each reporter line. Bars are the mean of *n* = 3 independent biological replicates; error bars are the SD of the three biological replicates; open circles are the individual biological replicate values, each the mean of two technical replicate wells. Source data are provided as a Source Data file.

To delineate the contributions of individual CDR loops, we projected them onto the peptide-MHC interface and mapped contact footprints. Although both complexes adopt a similar overall docking configuration, with an RMSD of 1.13 Å (Fig. 5a, b), the CDR3α and CDR3β loops display divergent engagement patterns (Fig. 5c, d, g). While both TCRs share the same CDR1β and CDR2β sequences, the four amino acid differences in their CDR3β loops-AGDL in TCR_pub_ (Fig. 5e and Supplementary Fig. 3a) and WTGV in TCR_priv_ (Fig. 5h)—produce distinct interactions that, together with CDR3α, shape the differential recognition described above. A key distinction lies at position 94: Trp94 in TCR_priv_ mediates dual contacts with Leu8 of the peptide and Thr71 of the MHC (Fig. 5h, i), whereas its replacement by Ala94 in TCR_pub_ eliminates these interactions (Fig. 5e, f) and instead supports the peptide-centric AGDL docking mode. TCR_pub_ interactions are primarily concentrated on the α2 helix (Fig. 5f), whereas TCR_priv_ engages both the α1 and α2 helices more evenly **(**Fig. 5i**)**. These findings provide a structural framework by which a combination of shared and unique CDR sequences results in differential peptide and MHC recognition.

### Alanine-scanning mutagenesis validates distinct recognition modes

To determine how peptide recognition by TCR_pub_ and TCR_priv_ depends on specific amino acid residues, we performed alanine-scanning mutagenesis across the ORF3a_(207-215)_ epitope and measured T cell activation in a co-culture assay. Although both clonotypes recognised the wild-type peptide efficiently, their sensitivity to individual substitutions differed. TCR_pub_ was affected by alanine substitutions across more positions of the peptide, indicating a greater dependence on peptide side chains for productive recognition. In particular, substitution at Asp4 caused a marked reduction in TCR_pub_ activation, whereas substitutions at Asp4 and, to a lesser extent, at Gln7 were comparatively well tolerated by TCR_priv_. More broadly, TCR_priv_ retained activity across a wider range of peptide variants, consistent with greater tolerance to sequence perturbation. These findings corroborate the structural analysis: TCR_pub_ adopts a more peptide-focused binding mode, whereas TCR_priv_ engages the pMHC through a more distributed interaction footprint (Fig. 5j and Supplementary Fig. 7).

### Structural context within the class I TCR/pMHC landscape

**Fig. 6:**
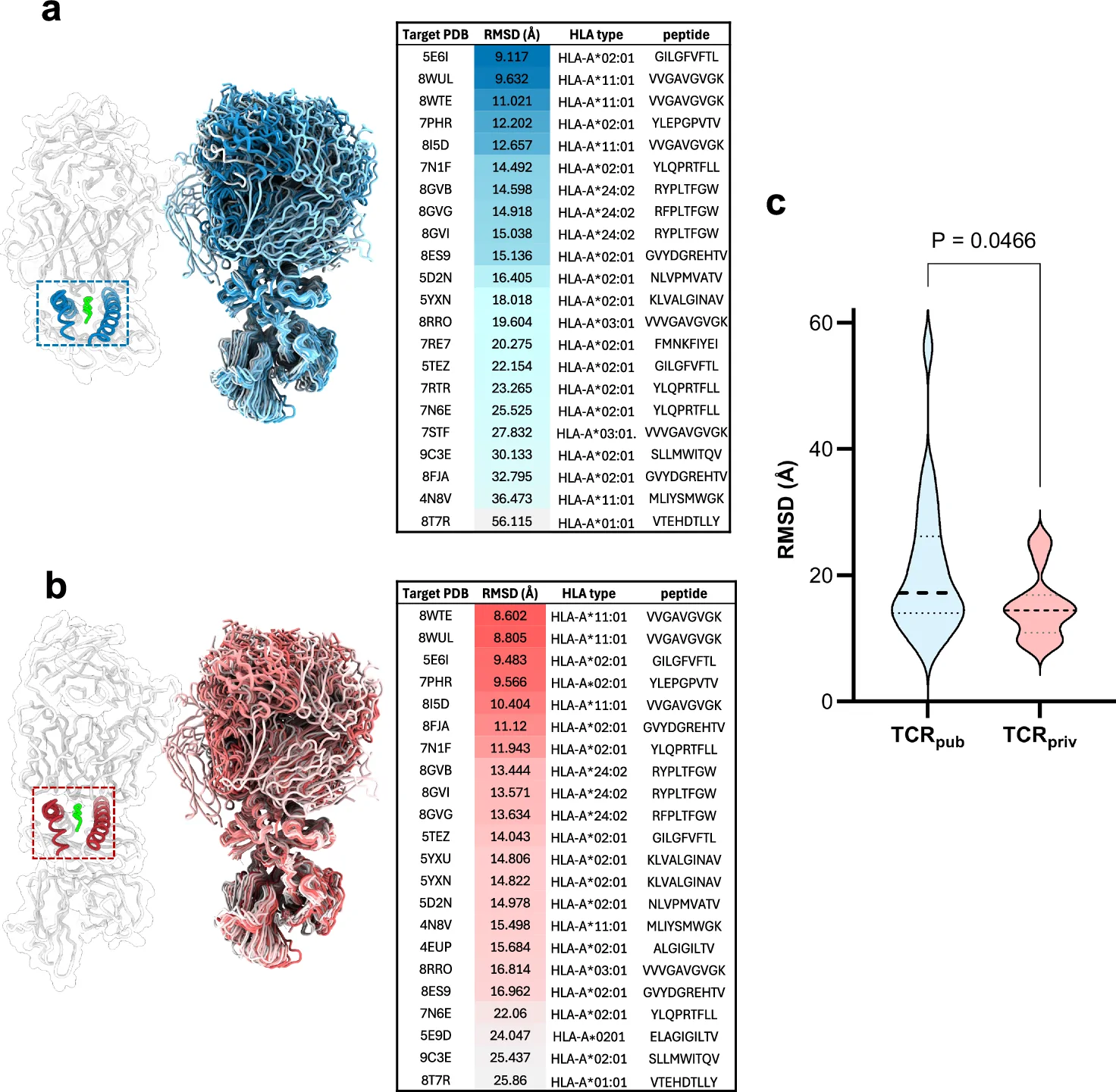
Structural flexibility of TCR/pMHC complexes. **a** Structural alignment of the TCR_pub_/pMHC complex against 1,388 class I TCR/pMHC structures from the TCR3d database. TM-align was used to identify structures with the highest topological similarity based on TM-scores. The top hits were superimposed on the MHC α1 and α2 helices (highlighted in red boxes, left panel) and the bound peptide. RMSD values for the TCR α and β chains were calculated in ChimeraX. **b** The same strategy was applied to the TCR_priv_/pMHC complex to obtain RMSD values for its TCR α and β chains. **c** Comparison of RMSD values for TCR_pub_/pMHC (*n*  =  50) and TCR_pub_/pMHC based on the reference structures. Data are shown as medians. A two-tailed Mann–Whitney test indicated a significant difference between the two groups (*U * =  157, *p * =  0.0466; *p  <  0.05). Median RMSD values: for TCR_pub_/pMHC (*n * =  50) and TCR_pub_/pMHC = 17.21, TCR_priv_/pMHC = 14.42. Hodges–Lehmann difference: –3.693 (95.07% CI: –8.287 to –0.0660). Source data are provided as a Source Data file.

To assess the structural variability of antigen recognition, we compared the TCR_pub_/pMHC and TCR_priv_/pMHC complexes against a representative set of class I TCR/pMHC structures from the TCR3d database^39^, selected on the basis of TM score^40^ and restricted to HLA-A alleles to maintain the MHC background. The TM-score, which ranges from 0 to 1 (with values above 0.5 generally indicating similar overall folds), is a length-independent metric that evaluates global topological similarity between two protein structures and is less sensitive to local deviations compared with RMSD (see Methods). TCR_priv_/pMHC exhibited relatively greater structural similarity to previously characterised complexes, as reflected by lower RMSD values, consistent with a more canonical binding mode. Conversely, TCR_pub_/pMHC showed higher RMSD values relative to the reference set, indicating a more divergent docking geometry. Overall, TCR_pub_/pMHC preserved the general architecture of class I TCR/pMHC complexes but displayed greater local variability at the TCR/pMHC interface and within the pMHC α1 and α2 domains (Fig. 6). These observations from a single public-private pair suggest that public TCRs may adopt CDR engagement modes that favour structural complementarity, although broader surveys across additional TCR/pMHC systems will be needed to establish the generality of this pattern.

## Discussion

Understanding how TCRs decode pMHCs is central to explaining the balance between diversity and reproducibility in antiviral immunity^3^. Although public TCRs are repeatedly observed across unrelated individuals, the structural logic that enables these clonotypes to emerge with such convergence remains unclear. By resolving near-native structures of two wild-type TCRs recognizing the same SARS-CoV-2 ORF3a_(207-215)_/HLA-A*01:01 complex, we reveal how closely related clonotypes, sharing an almost identical β chain, can nevertheless adopt distinct docking solutions that differentially partition peptide and MHC contacts. These findings provide an example for how conserved β-chain scaffolds can support multiple α-chain-dependent recognition modes, offering further insight into the emergence of public TCRs.

Recent advances in cryo-EM have resolved full-length TCRαβ-CD3, TCR/pMHC-co-receptor, and TCRγδ-CD3 assemblies^41–44^. In some cases, these studies have utilized affinity-enhanced constructs or stabilized elements to facilitate structure determination. In this context, our results indicate that cryo-EM can also be applied to wild-type CDR sequences to resolve TCR/pMHC complexes with micromolar affinity. More broadly, cryo-EM represents a complementary approach to crystallography for studying TCR recognition, particularly for complexes that are difficult to crystallize. Extending this approach to additional TCR-pMHC systems will be important for expanding a more comprehensive and less biased structural dataset.

Here, we report cryo-EM structures of two wild-type sequence TCRs in complex with the SARS-CoV-2 ORF3a_(207-215)_ epitope presented by HLA-A*01:01. These structures revealed how nearly identical TRBV5-1 β chains pair with distinct α chains (TRAV25 in TCR_priv_, TRAV3 in TCR_pub_), resulting in private and public TCR clonotypes. Both receptors engage the same peptide-MHC ligand with comparable functional avidity and micromolar binding affinity, demonstrating that different clonotypic architectures can mediate equally effective antiviral responses. Clonotype-specific CDR loop rearrangements further shape distinct binding geometries, illustrating how TCR diversity accommodates a single antigen through multiple docking modes and thereby expands the functional repertoire of the T cell response.

A central observation from our work is that TCR_pub_ engages the pMHC through a peptide-focused recognition strategy, whereas TCR_priv_ distributes its footprint more broadly across both the peptide and MHC α-helices via a canonical binding interface. These distinct recognition solutions arise despite similar overall docking geometry and comparable micromolar affinities, demonstrating that public versus private behaviour cannot be inferred from sequence or affinity alone. In fact, these differences arise from the combined contributions of both CDR3α and CDR3β loops despite the shared TRBV5-1 bias.

At a repertoire level, the TRBV5-1/CDR3β sequence of TCR_pub_ pairs with diverse α chains in various contexts, whereas the TRAV25 chain of TCR_priv_ remains uniquely linked to its β partner. In contrast, TRAV3-like α chains of TCR_pub_ are broadly distributed across many β chains. These patterns indicate convergent antigen-driven selection and frequent recombination of public TCR motifs within the repertoire. Consistent with these observations, analysis of the COVID-19 OTS dataset revealed distinct repertoire features between TCR_priv_ and TCR_pub_. Although all α-β similarity scores remained below the 0.8 threshold, TCR_priv_-associated sequences exhibited broader combinatorial diversity, consistent with a private, individual-specific response. In contrast, TCR_pub_-associated sequences displayed more conserved α-β pairings, suggesting recurrent usage of similar α-β combinations in multiple donors, a defining feature of public clonotypes. It should be noted that the 0.8 threshold is an operational cutoff reflecting ~2 amino acid differences, and even “private” TCRs may eventually appear in multiple individuals if sufficiently large population datasets are examined. These observations suggest that repertoire architecture and pairing diversity, rather than absolute sequence similarity, are key determinants distinguishing private and public TCR responses in COVID-19 (Fig. 2a, b). Furthermore, TRAV25/TRBV5-1 clonotypes occur in population-scale repertoires but lack structural characterization.

Similar patterns, where a dominant α chain pairs with multiple diverse β chains encompassing both public and private clonotypes, were also observed in our recent study^21^. Although these analyses were based on bulk β-chain sequencing and paired naive datasets remain limited, such findings suggest that certain public sequences pre-exist in the naive pool and are preferentially expanded upon antigen exposure^21,45^. Future studies combining single-cell paired repertoire sequencing with structural and functional analyses will be valuable to clarify how public and private TCRs are generated, selected, and maintained across individuals. Extending this approach to additional public and private TCR/pMHC pairs across diverse HLA alleles will systematically map the TCR conformational landscape. Integrating high-resolution structures with deep sequencing repertoires and biophysical profiling promises predictive models of TCR specificity and cross-reactivity, potentially informing next-generation adoptive T cell therapies.

## Methods

### Study participants and ethics

Patients were recruited from the John Radcliffe Hospital in Oxford, UK, between March 2020 and September 2021 by identification of patients hospitalized during the SARS-CoV-2 pandemic and recruited into the Sepsis Immunomics study. Blood samples were collected at least 28 days after symptom onset during the primary infection as described previously^46^. Written informed consent was obtained from all patients. Ethical approval was given by the South Central-Oxford C Research Ethics Committee in England (ref. ^19^/SC/0296).

### IFN-γ ELISpot assay

Ex vivo IFN-γ ELISpot assays were performed using either freshly isolated or cryopreserved PBMCs, or antigen-specific T cell clones, as previously described^46^. For ex vivo assays, peptides (Genscript, UK) were added at a concentration of 2 μg ml^−1^ to 2 × 10^5^ PBMCs per well and incubated for 16–18 h. Negative control wells were set up without peptide stimulation. For assays using T cell clones, autologous EBV-transformed B cell lines (BCLs) were pre-pulsed with peptides at fivefold titrated concentrations and subsequently co-cultured with T cells at an effector-to-target (E: T) ratio of 1:50 for at least 6 h. Co-cultures of EBV-transformed B cells and T cell clones without peptide stimulation were included as negative controls. Antigen-specific responses were quantified using AID ELISpot 7.0 software. The mean spot number from negative control wells was subtracted from peptide-stimulated wells, and results were expressed as spot-forming units (s.f.u.) per 10⁶ PBMCs for ex vivo ELISpot or s.f.u. per 400 T cells when using T cell clones. Responses were considered positive if the spot count was at least three times the mean of the negative control wells. Assays were excluded from further analysis if background responses in negative control wells exceeded 30 s.f.u. per 10^6^ PBMCs or per 400 T cells, or if positive control wells did not yield a measurable response.

### Single-cell sorting of ORF3a_(207-215)_ -A*01:01-specific CD8^+^ T cells

ORF3a_(207-215)_-HLA-A*01:01-specific CD8^+^ T cells were stained with a phycoerythrin (PE)-conjugated HLA-A*01:01 ORF3a_(207-215)_ pentamer (ProImmune). Nonviable cells were excluded using Live/Dead Fixable Aqua dye (Invitrogen). After washing, cells were stained with CD3-FITC (clone SK7, BD Biosciences, catalogue no. 345763; 1:25 dilution), CD8-PerCP-Cy5.5 (clone SK1, BD Biosciences, catalogue no. 565310; 1:33 dilution), CD14-BV510 (clone 63D3, BioLegend, catalogue no. 367124; 1:50 dilution), CD19-BV510 (clone HIB19, BioLegend, catalogue no. 302242; 1:50 dilution), and CD16-BV510 (clone 3G8, BioLegend, catalogue no. 302048; 1:50 dilution). Following exclusion of nonviable and CD14^+^, CD16^+^, and CD19^+^ cells, CD3^+^ CD8^+^ pentamer⁺ cells were sorted directly into 96-well PCR plates (Thermo Fisher Scientific) using a BD FACS Fusion or BD FACSAria III sorter (BD Biosciences) and stored at −80 °C for subsequent analyses.

### SmartSeq2 scRNA-seq and data processing

ScRNA-seq with ex vivo sorted CD8^+^ pentamer^+^ T cells (Proimmune, UK) was performed using SmartSeq2^47^ with the following modifications: reverse-transcription (RT) and PCR amplification were performed as previously described^47^ with the exception of using an ISPCR primer with biotin tagged at the 5′ end and increasing the number of cycles to 25. Sequencing libraries were prepared using the Nextera XT Library Preparation Kit (Illumina), and sequencing was performed on the Illumina NextSeq sequencing platform with NextSeq Control Software v.4. BCL files were converted to FASTQ format using bcl2fastq v.2.20.0.422 (Illumina). FASTQ files were aligned to the human genome hg19 using STAR v.2.6.1d^48–50^.

### SmartSeq2 TCR repertoire analysis

TCR sequences were reconstructed from scRNA-seq FASTQ files using MiXCR v.3.0.13^51,52^ generating separate TRA and TRB output files for downstream analysis. These output files were imported into R using the tcR package v.2.3.2. For paired αβ TCRs, cells were filtered to retain either one α and one β chain (1α1β) or two α chains and one β chain (2α1β). Additionally, lists of all cells expressing one β chain (regardless of the number of α chains) were generated for downstream analysis.

### Deep sequencing of TCR repertoire of T cell clones

T cell clones were generated by sorting CD8^+^ pentamer^+^ T cells into 96-well Tissue culture plates at single-cell level from thawed PBMCs. T cell clones were then expanded and maintained with irradiated allogeneic PBMCs every 2-3 weeks as described previously^53^. Purity and antigen specificity of T cell clones was checked by staining with peptide-MHC tetramers. T cell clones were included in the experiments only if their purity exceeded 95%. From each T cell clone, 1 × 10^5^ cells were harvested and washed with phosphate-buffered saline (PBS). Total RNA was extracted using RNeasy Plus Micro Kit (QIAGEN), and 100 ng of total RNA from each T cell clone was used to generate full-length TCR repertoire libraries for Illumina Sequencing using a SMARTer Human TCR a/b Profiling Kit (Takara) following the supplier’s instructions. The cDNA sequences corresponding to variable regions of TCRα and/or TCRβ transcripts were amplified with primers, including Illumina indices, allowing for sample barcoding. PCR products were then purified using AMPure beads (Beckman Coulter). The quantity and quality of cDNA libraries were checked on an Agilent 2100 Bioanalyzer system. Sequencing was performed using MiSeq reagent Kit v.3 (600 cycles) on MiSeq (Illumina) with MiSeq Control Software v.2.6.2.1. BCL files were converted to FASTQ files as described earlier. TCRs were extracted using MiXCR, and the resulting output files (TRA and TRB) were parsed into R using tcR as described earlier. TCRs were filtered to retain 1α1β for each clone^54^.

### Synthesis of peptide-MHC complex

4 mg of the peptide FTSDYYQLY (synthesized by Genscript, 90% purity) was used. The HLA-A*01:01 heavy chain cDNA was modified by replacing the transmembrane and cytosolic regions with a sequence encoding the BirA biotinylation enzyme recognition site, as previously described^55^. These modified heavy chains (amino acids 1–276) and β2-microglobulin were expressed in a prokaryotic system (pET; R&D Systems), purified from bacterial inclusion bodies, and solubilized in 8 M urea. Refolding was carried out by dilution in the presence of peptide at 4 °C for 48 h. The refolded complexes were concentrated using an Amicon Stirred Cell with a 10 kDa ultrafiltration membrane (Millipore), then desalted on a PD-10 column (Cytiva) prior to biotinylation with BirA (Avidity). Monomeric complexes were further purified by gel filtration chromatography (Superdex 75, Cytiva), with the 47 kDa peak collected and concentrated to 1 mg/mL using an Amicon Ultra centrifugal filter (10 kDa cutoff, Millipore).

### Expression and purification of soluble TCRs

Synthetic dsDNA fragments encoding TCRα and TCRβ chains were obtained from Twist Bioscience, with overlaps designed for Gibson assembly into the pD649 vector (Addgene plasmid #156543). Vectors were linearised with restriction enzymes and assembled using Gibson Isothermal Assembly (NEB). Assembled plasmids were transformed into *E. coli* DH5α (NEB C2987H) and plated on LB agar containing 1% carbenicillin. Colonies were cultured overnight in LB medium, and plasmids were extracted and verified by Sanger sequencing (GENEWIZ).

Recombinant TCRs were expressed in Expi293F cells (Thermo Fisher) maintained at 37 °C with 5% CO_2_, shaking at 160 rpm. On the day of transfection, cells were diluted to 3.0 × 10⁶ cells/mL in Expi293 medium (Thermo Fisher A1435101). Cells were transiently co-transfected with 25 µg of each TCRα and TCRβ plasmid using 150 µL of ExpiFectamine 293 reagent (Thermo Fisher), following the manufacturer’s protocol. At 18 h post-transfection, enhancers 1 and 2 were added. Supernatants were harvested on day 5, clarified by centrifugation (3200 g, 10 min, 4°C), and diluted 1:1 in HBS (30 mM HEPES pH 7.4, 150 mM NaCl). The buffer was supplemented with 20 mM Tris-HCl (pH 8.0) and 0.05% NaN₃. Proteins were captured by incubation with 3 mL of 50% Ni-NTA agarose (Qiagen 30210) overnight at 4 °C with gentle agitation.

His-tagged TCRs were eluted by IMAC, concentrated to ~500 µL, and treated overnight with HRV 3 C protease (ab285999) to remove tags and leucine zippers. Proteins were further purified by size-exclusion chromatography (SEC) on a Superdex 200 Increase 10/300 GL column (Cytiva) using an ÄKTA Pure system equilibrated with HBS. Fractions were analysed by SDS-PAGE, and those with correct size and purity were pooled, concentrated (Amicon Ultra-4, 100 kDa MWCO, Sigma-Aldrich UFC8100), aliquoted, and flash-frozen in liquid nitrogen for storage at −80 °C.

### T cell stimulation assay using NFAT-Luciferase reporter system

TCR constructs of TCR_priv_ and TCR_pub_ were generated by synthesising the TCR α and β coding sequences, each containing native signal peptides, and linking them via a P2A self-cleaving peptide. The fragments were designed with compatible overhangs and cloned into the pHR vector using MluI and BamHI restriction sites. The HLA-A*01:01 heavy chain sequence, including its native signal peptide, was cloned into the pHR vector using MluI and NotI restriction sites. Insert-vector assembly was performed using In-Fusion (ITA) cloning. Recombinant plasmids were transformed into DH5α *E. coli* (NEB C2987H) and plated on LB agar supplemented with 1 µg/mL carbenicillin. Single colonies were expanded in LB medium containing 1 µg/mL carbenicillin, and plasmid DNA was isolated and verified by Sanger sequencing.

For lentivirus production, HEK293T cells were cultured in DMEM medium supplemented with 10% FBS, 1% GlutaMAX, and 1% penicillin-streptomycin and seeded at 3 × 10⁵ cells per well in six-well plates 24 h before transfection. Cells were transfected with 750 ng transfer plasmid, 500 ng psPAX2, and 260 ng pMD2.G using 4.5 µL FuGENE (Promega) diluted in 100 µL Opti-MEM, following a 20 min incubation at room temperature. Prior to transfection, the culture medium was replaced with fresh RPMI medium supplemented with 10% FBS, 1% GlutaMAX, 1% penicillin-streptomycin, 1% HEPES, and 1% sodium pyruvate. The transfection mixture was added dropwise to each well, and plates were gently agitated before incubation at 37 °C with 5% CO₂. Lentiviral supernatants were collected 72 h post-transfection by centrifugation at 3500 rpm for 10 min at 4 °C.

For cell line generation, 1 × 10⁶ Jurkat CD8⁺ NFAT-luciferase reporter cells were transduced with 1 mL lentivirus encoding either TCR_priv_ or TCR_pub_. In parallel, 1 × 10⁶ T2 cells were transduced with 1 mL lentivirus encoding the HLA-A*01:01 heavy chain, assembled with endogenous β2-microglobulin. All cell lines were maintained in RPMI medium and cultured at 37 °C with 5% CO₂. Cells were allowed to recover for 1-week post-transduction before expansion and were seeded at 0.5 × 10⁶ cells/mL 24 h before co-culture assays. T2-A1 cells were resuspended at 0.25 × 10⁶ cells/mL in FBS-free RPMI medium supplemented with 1% GlutaMAX, 1% penicillin-streptomycin, 1% HEPES, and 1% sodium pyruvate, and 20 µL (5 × 10³ cells) were dispensed into each well of a 384-well flat-bottom plate. Peptides were prepared at final concentrations of 10 or 1 µM in FBS-free RPMI medium in a separate 96-well U-bottom plate, and 20 µL were subsequently added to the cells. Cells without peptide were prepared in parallel under identical conditions. Cells were incubated with peptide for 2 h at 37 °C, washed once by centrifugation at 1300 rpm for 3 min, and then co-cultured with 1 × 10⁴ Jurkat CD8⁺ NFAT-luciferase reporter cells expressing either TCR_priv_ or TCR_pub_. Co-cultures were maintained for 20 h at 37 °C. For assay readout, 20 µL Steady-Glo Luciferase substrate (Promega) was added to each well and incubated at room temperature for 10 min, and luminescence was measured using a CLARIOstar plate reader in Firefly Green luciferase mode as a readout of T cell activation.

### Surface plasmon resonance

SPR measurements were carried out on a Biacore S200 system (Cytiva). Biotinylated ORF3a_(207-215)_-HLA-A*01:01 complex was immobilised on a CAP sensor chip using the Biotin CAPture Kit (Cytiva), yielding 150-250 RU. An irrelevant control protein was immobilised on a reference flow cell at a similar level. Serial twofold dilutions of soluble 3C-cleaved TCRs were injected at 20 μL/min in HBS-P+ running buffer (10 mM HEPES, 150 mM NaCl, 0.05% v/v Surfactant P20, pH 7.4) at 37 °C. Binding responses at equilibrium were used to derive *K*_D_ values by fitting to a 1:1 Langmuir binding model using Biacore evaluation software. All measurements were performed in triplicate with alternating TCR injection order.

### Mass photometry analysis of TCR/pMHC complexes

Mass photometry measurements were performed at room temperature using a Refeyn TwoMP instrument (Refeyn Ltd, Oxford, UK). Prior to use, glass coverslips and silicone gaskets were thoroughly cleaned with HPLC-grade water followed by isopropanol, and dried under filtered air. TCR_priv_/pMHC and TCR_pub_/pMHC complexes were diluted to a final concentration of 200 nM in size-exclusion chromatography (SEC) buffer. To set the focal plane, 18 μl of buffer was first applied to the coverslip. Subsequently, 2 μl of the sample was added, and data acquisition was initiated using medium camera image settings. Each acquisition consisted of a 1-min movie, which was processed using ratiometric contrast analysis. Instrument calibration was carried out using reference standards including monomeric bovine serum albumin (66 kDa), dimeric BSA (132 kDa), and thyroglobulin (660 kDa), yielding a molecular weight accuracy within 5%. Experiments were independently repeated twice. Data were analyzed using DiscoverMP version 2.3 (Refeyn Ltd, Oxford, UK).

### Cryo-EM SPA sample preparation and data collection

Cryo-EM grids of TCR_priv_/pMHC and TCR_pub_/pMHC complexes were prepared by directly freezing the purified complexes at ~0.5–1.0 mg/mL onto glow-discharged Quantifoil R1.2/1.3 Cu 300 mesh grids (Quantifoil Micro Tools GmbH) using a Vitrobot Mark IV (Thermo Fisher Scientific), without chemical crosslinking. Grids were blotted for 3.0 s at room temperature and 100% humidity, then plunge-frozen into liquid ethane. Grid quality was initially screened on a Thermo Scientific Glacios transmission electron microscope. Final data collection was performed on a Titan Krios transmission electron microscope (Thermo Fisher Scientific) operated at 300 kV. The TCR_pub_/pMHC complex was imaged using a Gatan K3 direct electron detector, while the TCR_priv_/pMHC complex was collected on a Falcon 4i direct electron detector equipped with a Selectris X energy filter in EER mode. The total electron dose was 50 e⁻/Å², and images were collected with a defocus range of −0.8 to −2.5 μm. Micrographs were collected at a calibrated pixel size of 0.825 Å for the TCR_pub_/pMHC complex. In total, 12,657 and 10,222 movies were acquired for the TCR_pub_/pMHC (EMDB-54262, PDB 9RU5), TCR_priv_/pMHC (EMDB-54366, PDB 9RXM) (Supplementary Table 3).

### Cryo-EM single-particle analysis

All datasets were processed using CryoSparc v4.4.1^56^, following standard single-particle analysis workflows, including motion correction, CTF estimation, iterative classification, and refinement. For the TCR_pub_/pMHC dataset, 12,657 micrographs were initially processed using patch motion correction and CTF estimation. Initial particle coordinates were generated via blob picking, and Bin2 particle extraction yielded 5,535,316 particles. After several rounds of 2D classification, 70,035 particles were retained. A Topaz model was then trained on these and used to reprocess the dataset. Following Bin2 re-extraction and additional 2D classification, 899,071 particles were obtained and refined down to 422,641 particles through further classification. These particles were subjected to ab initio reconstruction and heterogeneous refinement, resulting in two major classes containing 207,209 and 166,747 particles, respectively. The 207,209-particle class was selected for downstream analysis. Particles were re-extracted at Bin1, and focused 3D classification was performed. For focused 3D classification and subsequent local refinement, a mask centred on the TCR–peptide–HLA-A*01:01 α1/α2 interface and the MHC β-sheet platform was used. The mask excluded the more flexible membrane-proximal MHC α3 domain and β2-microglobulin; therefore, these regions are absent from the final focused reconstruction used for model building. No manual deletion or post-refinement editing of map density was performed. Classes displaying well-defined α-helices were selected for non-uniform refinement, followed by local refinement, yielding a global resolution of 3.17 Å at the FSC 0.143 criterion. Final post-processing included map sharpening and local resolution estimation (Supplementary Fig. 4).

For the TCR_priv_/pMHC dataset, a total of 10,222 movies were subjected to patch motion correction and CTF estimation. Initial particle coordinates were obtained using Topaz extraction with a model pre-trained on the TCR_pub_/pMHC dataset. Bin2 particle extraction yielded 3,903,441 particles, which were refined through multiple rounds of 2D classification, resulting in 89,350 particles for initial non-uniform refinement. To improve data quality, Topaz was retrained on high-quality particles from the initial rounds, and a new particle set was extracted and re-extracted at Bin1. Subsequent 2D classification using ViewSelect retained 457,735 particles. These were imported into RELION-5 for 3D auto-refinement with Blush regularization, yielding a global resolution of 3 Å at the FSC 0.143 criterion. Final post-processing involved map sharpening and local resolution estimation (Supplementary Fig. 5).

### Model building, refinement, and structure visualization

Initial atomic models of TCRα, TCRβ, the ORF3a_(207-215)_ peptide, and HLA-A*01:01 heavy chain were generated using AlphaFold3 multimer predictions^57^. For the TCR_priv_/pMHC and TCR_pub_/pMHC monomeric complexes, models were rigid-body docked into cryo-EM densities and refined iteratively using manual adjustments in Coot^58^ and real-space refinement in Phenix^59^. Structural figures were prepared using UCSF ChimeraX^60^.

### Sequence analysis

TCR_priv_ and TCR_pub_ α- and β-chain sequences were aligned using MUSCLE (v3.8)^61^ with default parameters. The alignment was visualized using ESPript 3.0^62^, with annotated CDR loops, and contact residues (Supplementary Fig. 3a). Structural comparisons between TCR_priv_/pMHC and TCR_pub_/pMHC were performed in ChimeraX (v1.7), and Cα RMSD values were calculated using the MatchMaker tool (Supplementary Fig. 3b). To assess structural similarity, TCR_priv_/pMHC and TCR_pub_/pMHC were aligned to 1,388 class I TCR-pMHC structures from TCR3d database^39^ using TM-align^40^. Alignments were performed by superposition on the MHC α1-α2 helices. To ensure comparable MHC backgrounds, only HLA-A-restricted complexes were retained for subsequent analysis, yielding a representative subset used for TM-score ranking and RMSD comparison (Supplementary Table 4). The best structural matches were selected based on TM-score and visual inspection, and pairwise RMSD values (Cα atoms) between TCR chains were computed in UCSF ChimeraX^60^ (Fig. 6). TM-scores (ranging from 0 to 1) were used to quantify global structural similarity independent of sequence length.

OTS COVID TCR sequence data were obtained from the Oxford Protein Informatics Group (OPIG) COVID-19 TCR database (https://opig.stats.ox.ac.uk/webapps/ots/ots_paired). The CDR3 sequences of TCR_priv_ α/β and TCR_pub_ α/β were pre-processed by removing the leading cysteine and terminal phenylalanine residues. Pairwise sequence similarity between each of the TCR_priv_ and TCR_pub_ α- and β-chain of CDR3 regions and all CDR3 sequences in the COVID OTS dataset was calculated using the stringsimmatrix function from the stringdist R package (v0.9.15), based on Levenshtein distance score. Similarity scores ranged from 0 (no similarity) to 1 (identical sequences). A threshold of >0.8, corresponding to approximately two amino acid differences, was applied to define sequences as “similar.” TCRs with CDR3α or CDR3β regions showing a similarity score >0.8 to TCR_priv_ or TCR_pub_ were classified as TCR_priv_ α/β-similar or TCR_pub_ α/β-similar, respectively. Paired α-β chain associations of these similar TCRs were visualized using Circos (v0.69) to illustrate V gene usage and pairing diversity. Distinct colour segments represent V gene families (TRAV or TRBV), while grey connecting lines indicate α-β pairings (Fig. 2a, b and Supplementary Data 2).

### Quantification and statistical analysis

Statistical analyses were performed using GraphPad Prism (v10.3.0). EC_50_ values of T cell clones were calculated by nonlinear regression using a variable slope (four-parameter logistic) model in dose-response stimulation assays. Data are presented as mean ± SD or as medians, as indicated in the figure legends. Comparisons between two groups were performed using two-tailed Mann–Whitney tests. Effect sizes were estimated using the Hodges-Lehmann method with 95% confidence intervals. *P* values were corrected for multiple comparisons where applicable. Statistical significance is indicated as follows: *P ≤ 0.05, **P ≤ 0.005, ***P ≤ 0.0005, ****P ≤ 0.0001. Investigators were not blinded to group allocation during experiments or outcome assessment.

### Reporting summary

Further information on research design is available in the Nature Portfolio Reporting Summary linked to this article.

## Supplementary information

Supplementary Information

Description of Additional Supplementary Files

Supplementary Data 1

Reporting Summary

Transparent Peer Review file

Supplementary Data 2

## Source data

Source Data

## Data Availability

Cryo-EM maps and atomic models reported in this study have been deposited in the Electron Microscopy Data Bank (EMDB) and the RCSB Protein Data Bank (PDB). Accession codes and corresponding URLs are as follows: TCR_priv_/pMHC map EMD-54366 (https://www.ebi.ac.uk/emdb/EMD-54366) and model PDB 9RXM (https://www.rcsb.org/structure/9RXM); TCR_pub_/pMHC map EMD-54262 (https://www.ebi.ac.uk/emdb/EMD-54262) and model PDB 9RU5 (https://www.rcsb.org/structure/9RU5). All data are publicly available for download and visualization from the respective databases. All other data are available in the article and its Supplementary files or from the corresponding author upon request. Source data are provided with this paper.
