# Supplementary material for: Unveiling structural determinants of peptide recognition by public and private SARS-CoV-2-specific T-cell receptors
Source: Nat Commun. 2026 Sep 22;17:11026. doi: 10.1038/s41467-026-77827-z (PMC13687542; doi:10.1038/s41467-026-77827-z)
Supplement: Supplementary file 1 [file 41467_2026_77827_MOESM1_ESM.pdf]

Supplementary tables and figures

Supplementary Table 1. T cell response against epitope HLA-A*01:01 ORF3a$_{(207-215)}$ measured by *ex vivo* IFN-γ ELISpot. SFU: Spot Forming Unit from 18 HLA-A*01:01 positive individuals.

Participant ID	HLA-A*01:01	Responder	SFU/10^6PBMCs
C-COV19-001	No		
C-COV19-002	No		
C-COV19-003	No		
C-COV19-004	No		
C-COV19-005	Yes	Yes	25
C-COV19-006	Yes	Yes	30
C-COV19-007	Yes	Yes	25
C-COV19-008	No		
C-COV19-009	No		
C-COV19-010	No		
C-COV19-011	Yes	No	0
C-COV19-012	No		
C-COV19-014	Yes	Yes	15
C-COV19-018	No		
C-COV19-020	No		
C-COV19-021	No		
C-COV19-022	Yes	Yes	50
C-COV19-023	No		
C-COV19-024	No		
C-COV19-025	No		
C-COV19-026	No		
C-COV19-027	No		
C-COV19-028	No		
C-COV19-029	No		
C-COV19-030	No		
C-COV19-031	No		
C-COV19-032	No		
C-COV19-033	No		
C-COV19-034	Yes	Yes	35
C-COV19-035	No		
C-COV19-036	Yes	Yes	120
C-COV19-037	Yes	Yes	115
C-COV19-038	No		
C-COV19-039	Yes	Yes	60
C-COV19-040	Yes	Yes	90
C-COV19-041	Yes	Yes	125
C-COV19-042	No		
C-COV19-043	No		
C-COV19-044	No		
C-COV19-045	Yes	Yes	125
C-COV19-046	No		
C-COV19-047	No		
C-COV19-048	No		
C-COV19-050	No		
C-COV19-051	Yes	Yes	40
C-COV19-054	Yes	NO	0
C-COV19-055	No		
C-COV19-056	Yes	Yes	35
C-COV19-059	Yes	Yes	110
C-COV19-060	No		
C-COV19-061	No		
C-COV19-062	Yes	Yes	40

Supplementary Table 2. Clonotype and CDR3 amino acid sequences of TCR_{pub} and TCR_{priv}

ID	TRBV	CDR3_beta	TRAV	CDR3_alpha	TRAJ	Va_ public	Paired_ public
**TCR_{pub}**	TRBV5-1	CASSLAGDLGTEAFF	TRAV3	CAVRVVTSGGSYIPTF	TRAJ6	Public	Public
**TCR_{priv}**	TRBV5-1	CASSLWTGVGTEAFF	TRAV25	CASDREGGSEKLVF	TRAJ57	Private	Private

Supplementary Table 3. Cryo-EM data collection and structure determination of TCR/pMHC complexes

Data collection and processing		
Complex	**TCR_{pub}/pMHC**<br>EMDB-54262<br>PDB_ID: 9RU5	**TCR_{priv}/pMHC**<br>EMDB-54336<br>PDB_ID: 9RXM
Microscope	Titan Krios	
Voltage	300 kV	
Electron dose	50 e-/Å^2	
Detector	Gatan K3	Falcon<br>4i SelectrisX
Defocus Range	-0.8 to -2.5 μm	
Pixel Size	0.825	0.7303
Micrograph numbers	12657	10222
Particle numbers	5,535,316	3,903,441
Symmetry	C1	C1
Map resolution (Å)	3.4	3.37
FSC threshold	0.143	0.143
Local resolution range (Å)	2.92-11.21	2.5-9.67
**Refinement**		
Initial Model used (PDB code)	AlphaFold3 Prediction	
FSC threshold	0.143	0.143
Non-hydrogen atoms	4825	6063
Residues	605	752
Bond length(Å)	0.003	0.003
Bond angles(°)	0.699	0.642
MolProbity score	2.19	2.19
Clashscore	17.33	17.33
Rotamers Outliers (%)	0	0
Ramachandran		
Favored(%)	92.84	88.41
Allowed(%)	6.98	11.45
Outliers(%)	0.17	0.14

Supplementary Table 4 . Structural comparison and RMSD analysis of TCR_{pub} and TCR_{priv} complexes. This table contains the RMSD values of the PDB structures used in Figure 6c. HLA alleles highlighted in white correspond to the same subtype, while the others (in grey) represent different subtypes.

TCR_{pub} Chains	Target PDB	Target Chains	CA Atoms	RMSD (Å)	HLA type	peptide
A&B	5E6I	P&Q	233	9.117	HLA-A*02:01	GILGFVFTL
A&B	8WUL	C&D	237	9.632	HLA-A*11:01	VVGAVGVGK
A&B	6UON	G&H	235	10.192	HLA-C*08:02	GADGVGKSAL
A&B	8WTE	A&B	234	11.021	HLA-A*11:01	VVGAVGVGK
A&B	7PHR	A&B	236	12.202	HLA-A*02:01	YLEPGPVTV
A&B	8I5D	A&B	235	12.657	HLA-A*11:01	VVGAVGVGK
A&B	4MVB	C&D	236	13.854	H-2 L^d (mouse)	QPAEGGFQL
A&B	6BJ2	D&E	237	13.878	HLA-B*35:01	IPLTEEAEL
A&B	3RGV	A&B	236	13.99	H-2 K^b (mouse)	WIYVYRPMGCGGS
A&B	6AVF	A&B	236	14.142	HLA-B*07:02	APRGPHGGAASGL
A&B	4MS8	C&D	236	14.235	H-2 L^d (mouse)	SPAEAGFFL
A&B	7N1F	D&E	236	14.492	HLA-A*02:01	YLQPRTFLL
A&B	8GVB	A&B	228	14.598	HLA-A*24:02	RYPLTFGW
A&B	8GVG	A&B	235	14.918	HLA-A*24:02	RFPLTFGW
A&B	8GVI	A&B	235	15.038	HLA-A*24:02	RYPLTFGW
A&B	8ES9	A&B	236	15.136	HLA-A*02:01	GVYDGREHTV
A&B	5D2N	C&F	237	16.405	HLA-A*02:01	NLVPMVATV
A&B	6AVG	B&E	235	16.468	HLA-B*07:02	APRGPHGGAASGL
A&B	7N2S	D&F	236	16.689	HLA-B*27	TRLALIAPK
A&B	2E7L	B&C	119	16.783	H-2 L^d (mouse)	QLSPFPFDL
A&B	3TPU	A&B	237	16.905	H-2 L^d (mouse)	FLSPFWFDI
A&B	8D5Q	A&B	237	16.917	H-2 L^d (mouse)	HPGSVNEFDF
A&B	7N2N	D&F	236	17.058	HLA-B*27	TRLALIAPK
A&B	7N2R	D&F	236	17.598	HLA-B*27.	TRLALIAPK
A&B	4NHU	C&D	236	17.607	H-2 L^d (mouse)	GGGAPWNPAMMI
A&B	7N2P	D&F	236	17.919	HLA-B*27	GQVMVVAPR
A&B	5YXN	A&B	236	18.018	HLA-A*02:01	KLVALGINAV
A&B	7N2Q	D&F	236	18.521	HLA-B*27	LRVMMLAPF
A&B	2CKB	A&B	237	18.713	H-2 L^d (mouse)	EQYKFYSV
A&B	1G6R	A&B	237	19.231	H-2 K^b (mouse)	SIYRYYGL
A&B	1NAM	A&B	116	19.534	H-2 K^b (mouse)	RGYVYQGL
A&B	2OL3	A&B	118	19.575	H-2 K^b (mouse)	SQYYYNSL
A&B	1MWA	A&B	237	19.582	H-2 K^b (mouse)	EQYKFYSV
A&B	8RRO	A&B	235	19.604	HLA-A*03:01	VVVGAVGVGK
A&B	7RE7	H&L	217	20.275	HLA-A*02:01	FMNKFIYEI
A&B	7N2O	D&F	236	20.444	HLA-B*27	LRVMMLAPF
A&B	4MXQ	C&D	237	21.055	H-2 L^d (mouse)	SPAPRPLDL
A&B	5TEZ	I&J	235	22.154	HLA-A*02:01	GILGFVFTL
A&B	4N5E	C&D	237	22.341	H-2 L^d (mouse)	VPYMAEFGM
A&B	3MV9	D&E	226	22.906	HLA-B*35:01	HPVGEADYFEY
A&B	7RTR	D&E	225	23.265	HLA-A*02:01	YLQPRTFLL
A&B	7N6E	I&J	225	25.525	HLA-A*02:01	YLQPRTFLL
A&B	7JWJ	E&D	224	26.619	H-2 L^d (mouse)	ASNENMETM
A&B	7STF	H&L	221	27.832	HLA-A*03:01.	VVVGAVGVGK
A&B	8ENH	D&E	228	28.754	HLA-B*35:01	LPFEKSTIM
A&B	9C3E	A&B	217	30.133	HLA-A*02:01	SLLMWITQV
A&B	8FJA	E&D	221	32.795	HLA-A*02:01	GVYDGREHTV
A&B	3CII	H&G	170	35.608	HLA-E	VMAPRTLFL
A&B	4N8V	G	190	36.473	HLA-A*11:01	MLIYSMWGK
A&B	8T7R	I&J	225	56.115	HLA-A*01:01	VTEHDTLLY

TCR_{priv} Chains	Target PDB	Target Chains	CA Atoms	RMSD (Å)	HLA type	peptide
A&B	8WTE	C&D	221	8.602	HLA-A*11:01	VVGAVGVGK
A&B	8WUL	C&D	221	8.805	HLA-A*11:01	VVGAVGVGK
A&B	5E6I	G&H	219	9.483	HLA-A*02:01	GILGFVFTL
A&B	7PHR	A&B	220	9.566	HLA-A*02:01	YLEPGPVTV
A&B	6UON	G&H	214	9.892	HLA-C*08:02	GADGVGKSAL
A&B	8I5D	A&B	219	10.404	HLA-A*11:01	VVGAVGVGK
A&B	8FJA	A&B	220	11.12	HLA-A*02:01	GVYDGREHTV
A&B	3RGV	A&B	220	11.137	H-2 K^b (mouse)	WIYVYRPMGCGGS
A&B	7N1F	D&E	220	11.943	HLA-A*02:01	YLQPRTFLL
A&B	6BJ2	D&E	221	12.25	HLA-B*35:01	IPLTEEAEL
A&B	4MVB	C&D	220	12.386	H-2 L^d (mouse)	QPAEGGFQL
A&B	4MS8	C&D	220	13.426	H-2 L^d (mouse)	SPAEAGFFL
A&B	8GVB	A&B	212	13.444	HLA-A*24:02	RYPLTFGW
A&B	8GVI	A&B	219	13.571	HLA-A*24:02	RYPLTFGW
A&B	8GVG	A&B	219	13.634	HLA-A*24:02	RFPLTFGW
A&B	3MV7	D&E	115	13.872	HLA-B*35:01	HPVGEADYFEY
A&B	5TEZ	D&F	220	14.043	HLA-A*02:01	GILGFVFTL
A&B	3TPU	A&B	221	14.078	H-2 L^d (mouse)	FLSPFWFDI
A&B	7N2O	D&F	220	14.592	HLA-B*27	LRVMMLAPF
A&B	2E7L	B&C	119	14.621	H-2 L^d (mouse)	QLSPFPFDL
A&B	8D5Q	A&B	221	14.743	H-2 L^d (mouse)	HPGSVNEFDF
A&B	5YXU	A&B	221	14.806	HLA-A*02:01	KLVALGINAV
A&B	5YXN	A&B	220	14.822	HLA-A*02:01	KLVALGINAV
A&B	5D2N	C&F	221	14.978	HLA-A*02:01	NLVPMVATV
A&B	6AVF	B&E	219	14.979	HLA-B*07:02	APRGPHGGAASGL
A&B	7N2R	D&F	220	15.124	HLA-B*27	TRLALIAPK
A&B	4N8V	U&J	219	15.498	HLA-A*11:01	MLIYSMWGK
A&B	4EUP	D&F	220	15.684	HLA-A*02:01	ALGIGILTV
A&B	4NHU	C&D	220	15.772	H-2 L^d (mouse)	GGGAPWNPAMMI
A&B	7N2Q	D&F	220	16.1	HLA-B*27	LRVMMLAPF
A&B	2CKB	A&B	221	16.583	H-2 K^b (mouse)	EQYKFYSV
A&B	1NAM	A&B	116	16.602	H-2 K^b (mouse)	RGYVYQGL
A&B	8RRO	F&G	220	16.814	HLA-A*03:01	VVVGAVGVGK
A&B	8ES9	A&B	220	16.962	HLA-A*02:01	GVYDGREHTV
A&B	1G6R	A&B	221	17.059	H-2 K^b (mouse)	SIYRYYGL
A&B	2OL3	A&B	118	17.327	H-2 K^b (mouse)	SQYYYNSL
A&B	1MWA	A&B	221	17.439	H-2 K^b (mouse)	EQYKFYSV
A&B	6AVG	H&L	204	17.637	HLA-B*07:02	APRGPHGGAASGL
A&B	7N2S	D&F	220	18.41	HLA-B*27	TRLALIAPK
A&B	4MXQ	C&D	221	19.72	H-2 L^d (mouse)	SPAPRPLDL
A&B	4PRH	I&J	219	19.885	HLA-B*35:01	HPVGDADYFEY
A&B	4N5E	C&D	221	20.866	H-2 L^d (mouse)	VPYMAEFGM
A&B	7N2P	D&E	210	21.84	HLA-B*27	GQVMVVAPR
A&B	7N6E	D&E	210	22.06	HLA-A*02:01	YLQPRTFLL
A&B	5E9D	I&J	209	24.047	HLA-A*0201	ELAGIGILTV
A&B	9C3E	A&B	161	25.437	HLA-A*02:01	SLLMWITQV
A&B	8T7R	H&L	208	25.86	HLA-A*01:01	VTEHDTLLY
A&B	7STF	E&D	208	32.411	HLA-A*03:01	VVVGAVGVGK
A&B	7N2N	G	183	34.794	HLA-B*27	TRLALIAPK
A&B	7RE7	R&Q	210	56.516	HLA-A*02:01	FMNKFIYEI

Supplementary Table 5. Summary of key TCR–pMHC contacts identified in the cryo-EM structures of TCR_{pub} and TCR_{priv} complexes.

Complex	TCR Chain	CDR Loop	TCR Residue	Peptide/MHC Residue	Interaction Type
TCRpub/pMHC	β	CDR2β	Arg53	Asp4 (peptide)	Hydrogen bond
TCRpub/pMHC	β	CDR3β	Ala94	Tyr5 (peptide)	Hydrophobic
TCRpub/pMHC	α	CDR3α	Gly99	Gln153 (MHC α2 helix)	Hydrogen bond
TCRpub/pMHC	α	CDR3α	Val95	Val156 (MHC α2 helix)	Hydrophobic
TCRpub/pMHC	β	CDR2β	Thr51	Arg63 (MHC α1 helix)	Hydrophobic
TCRpub/pMHC	α	CDR1α	Tyr31	Arg155 (MHC α2 helix)	Hydrogen bond
TCRpriv/pMHC	β	CDR2β	Arg53	Tyr6 (peptide)	Hydrogen bond
TCRpriv/pMHC	β	CDR2β	Arg53	Asp4 (peptide)	Salt bridge
TCRpriv/pMHC	β	CDR3β	Trp94	Leu8 (peptide)	Hydrophobic
TCRpriv/pMHC	α	CDR3α	Glu93	Arg161 (MHC α2 helix)	Hydrogen bond
TCRpriv/pMHC	α	CDR2α	Gln47	Gln153 (MHC α2 helix)	Hydrogen bond
TCRpriv/pMHC	β	CDR2β	Asn54	Arg63 (MHC α1 helix)	Salt bridge
TCRpriv/pMHC	α	CDR1α	Thr27	Val156 (MHC α2 helix)	Hydrophobic

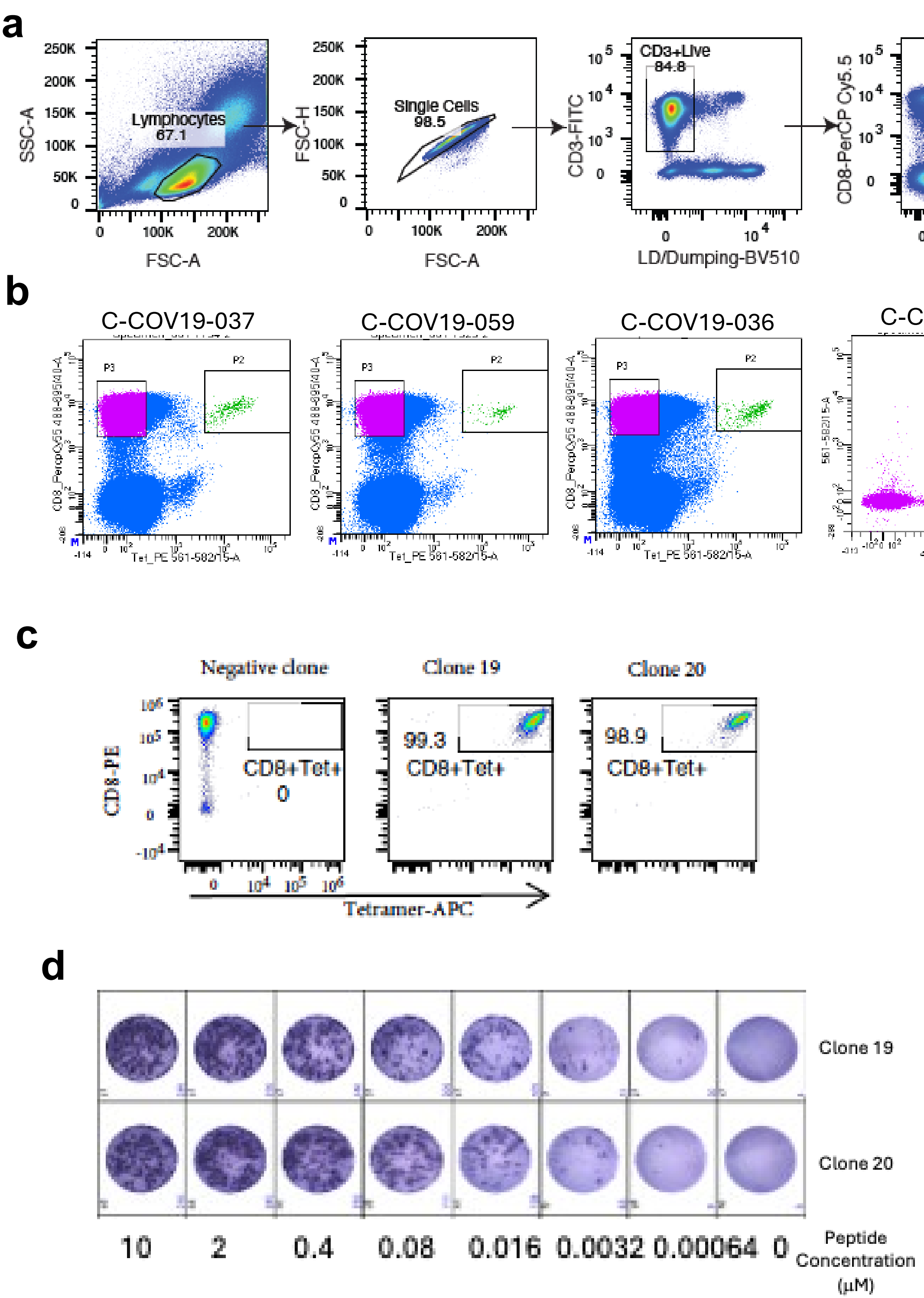

Supplementary Figure 1. Flow cytometry data. (**a**) Gating strategy used for flow cytometric sorting of HLA-A01:01/ORF3a$_{(207–215)}$-specific CD8^{+} T cells. (**b**) Representative FACS plots showing the sorted Pentamer^{+} cell populations. (**c**) HLA-A01:01/ORF3a$_{(207–215)}$-specific CD8^{+} T cells were single-cell sorted and expanded *in vitro* as T cell clones. The purity of the clones after expansion was confirmed by staining with peptide–MHC tetramers. (**d**) Representative ELISpot image from the EC$_{50}$ assay. T cell clones were co-cultured with autologous EBV-transformed B cell lines (BCLs) pre-loaded with peptides at fivefold serial dilutions starting from 10 µM.

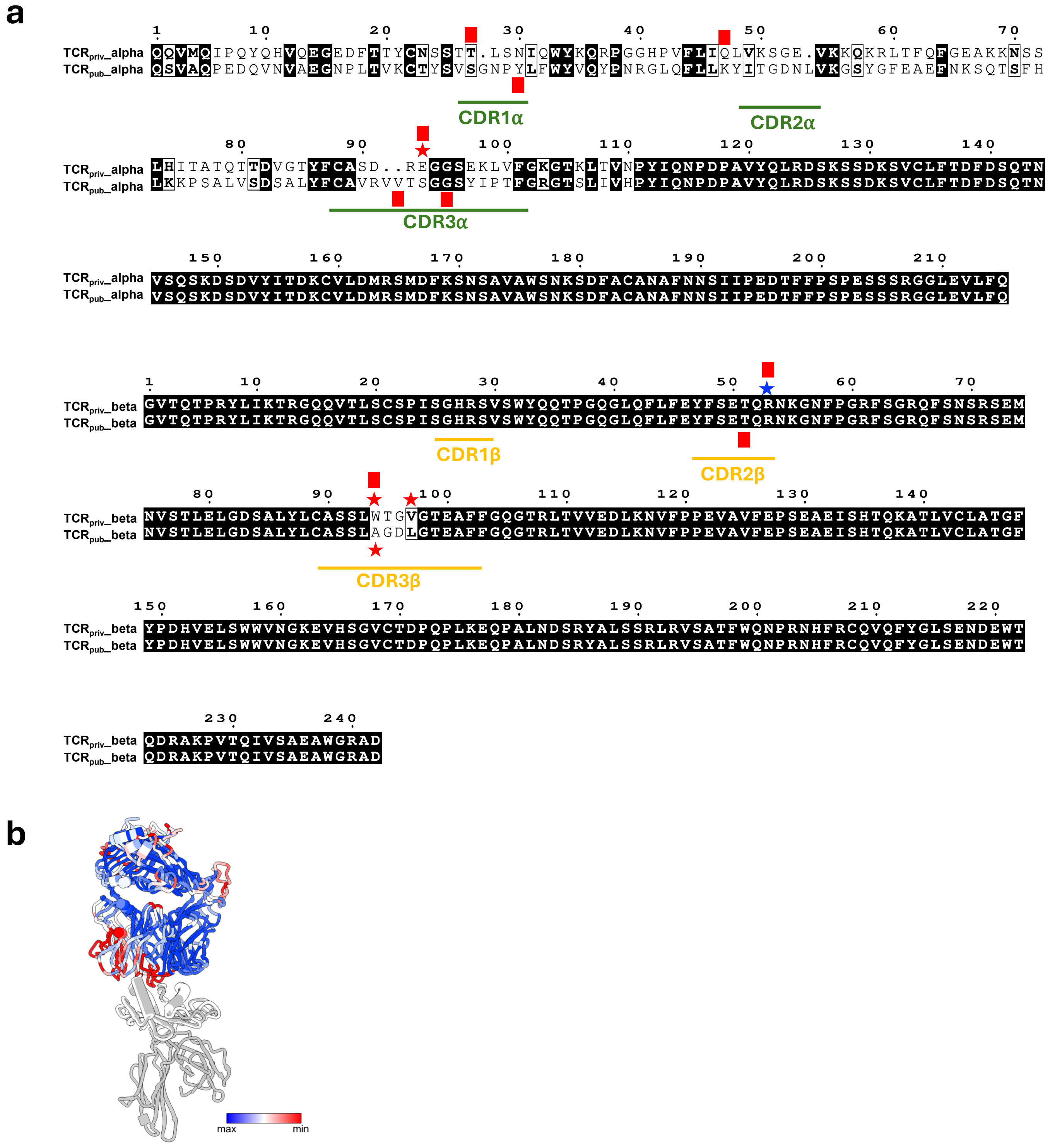

Supplementary Figure 2. (**a**) Sequence alignment of the TCR_{pub} and TCR_{priv} α (top) and β (bottom) chains. Key residues involved in peptide interaction are marked with asterisks: red asterisks indicate peptide-contacting residues specific to the α or β chain of TCR_{pub} or TCR_{priv}, while blue asterisks denote peptide-contacting residues shared between the α and β chains of both receptors. Residues involved in MHC interaction are boxed: red boxes indicate MHC-contacting residues specific to the α or β chain of TCR_{priv} or TCR_{priv}. CDR loops are colored by chain type (green, α; yellow, β). The sequence alignment was performed using MUSCLE and visualized with ESPript 3.0. (**b**) TCR_{pub} aligned with the TCR_{priv}, with an RMSD of 1.134 Å. Sequence alignments of TCR-pMHC structures are color-coded based on residue conservation: blue represents highly conserved residues, while red indicates the least conserved.

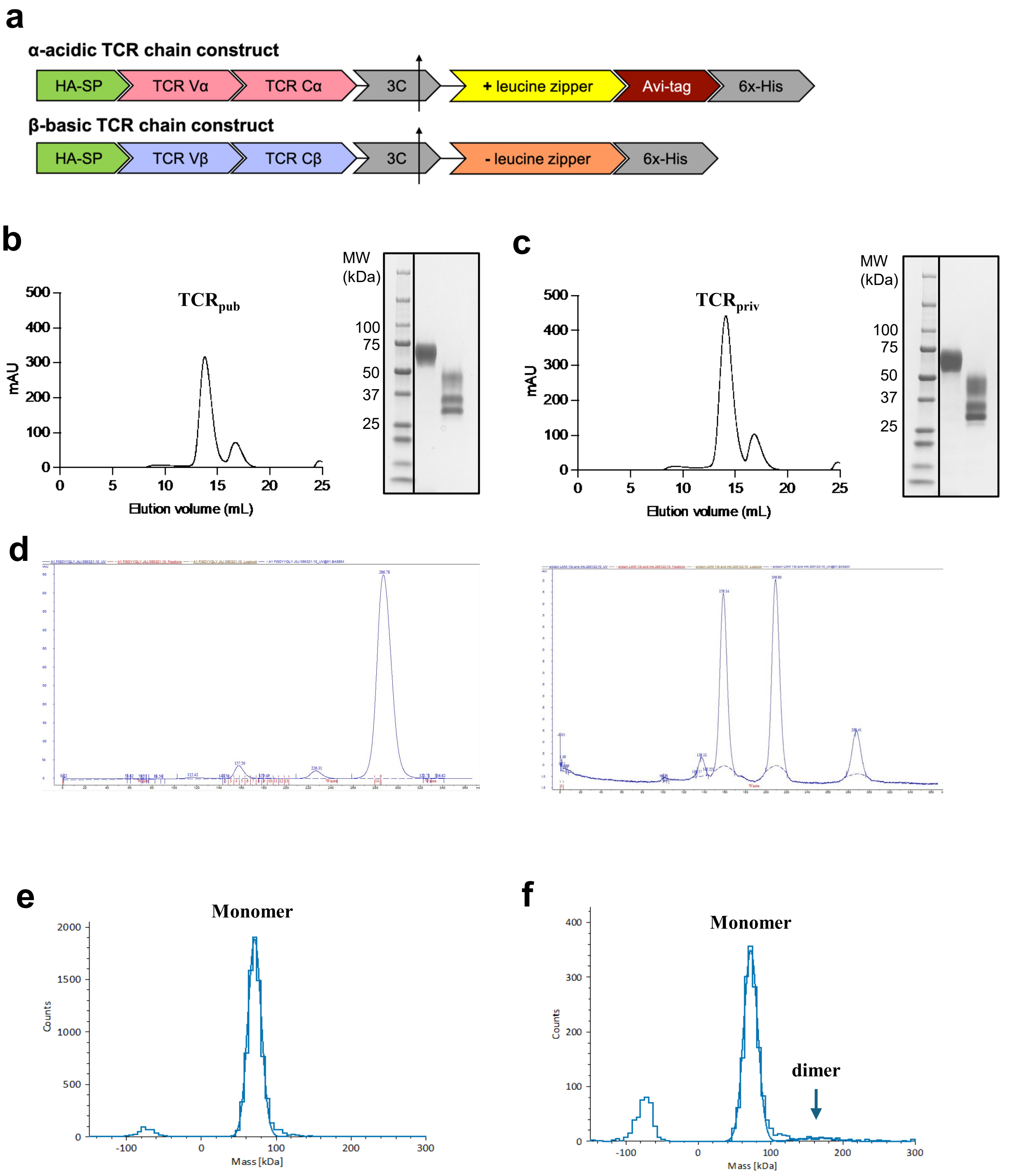

Supplementary Figure 3. Expression of soluble ORF3a$_{207\text{-}215}$-A*01:01 TCRs. **(a)** Schematic of a generic soluble TCRαβ DNA construct. **(b-c)** Size-exclusion chromatography profile (left) and SDS-PAGE analysis (right, under reducing and non-reducing conditions) of the 3C-treated soluble ORF3a-reactive TCR$_{pub}$ **(b)** and TCR$_{priv.}$ **(c).** The left peak eluting at approximately 14.0 mL corresponds to the ORF3a TCRs, and the right peak eluting after 16 mL corresponds to the cleaved zippers and tags. **(d)** Gel filtration chromatograms showing the purification of the refolded peptide–MHC complex (top) and the calibration standards (bottom). For calibration, ovalbumin (43 kDa) eluted at 158.14 mL and ribonuclease A (13.7 kDa) at 208.8 mL. The pMHC complex (47 kDa) eluted as a peak at 157.50 mL, whereas free β$_2$M (12 kDa) eluted at 226.31 mL.

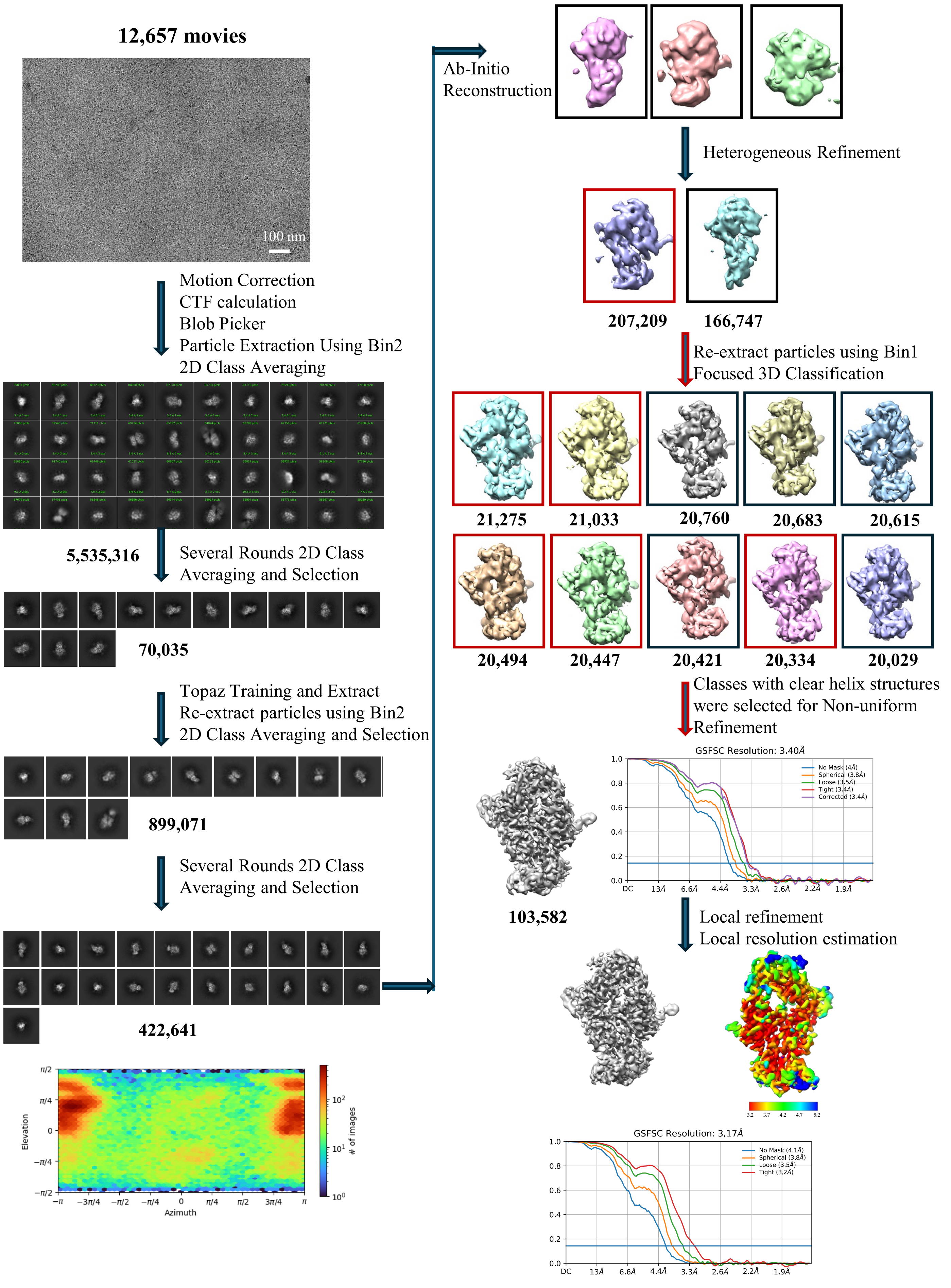

Supplementary Figure 4. Single particle cryo-EM data processing workflow for the TCR_{pub}/pMHC complex.

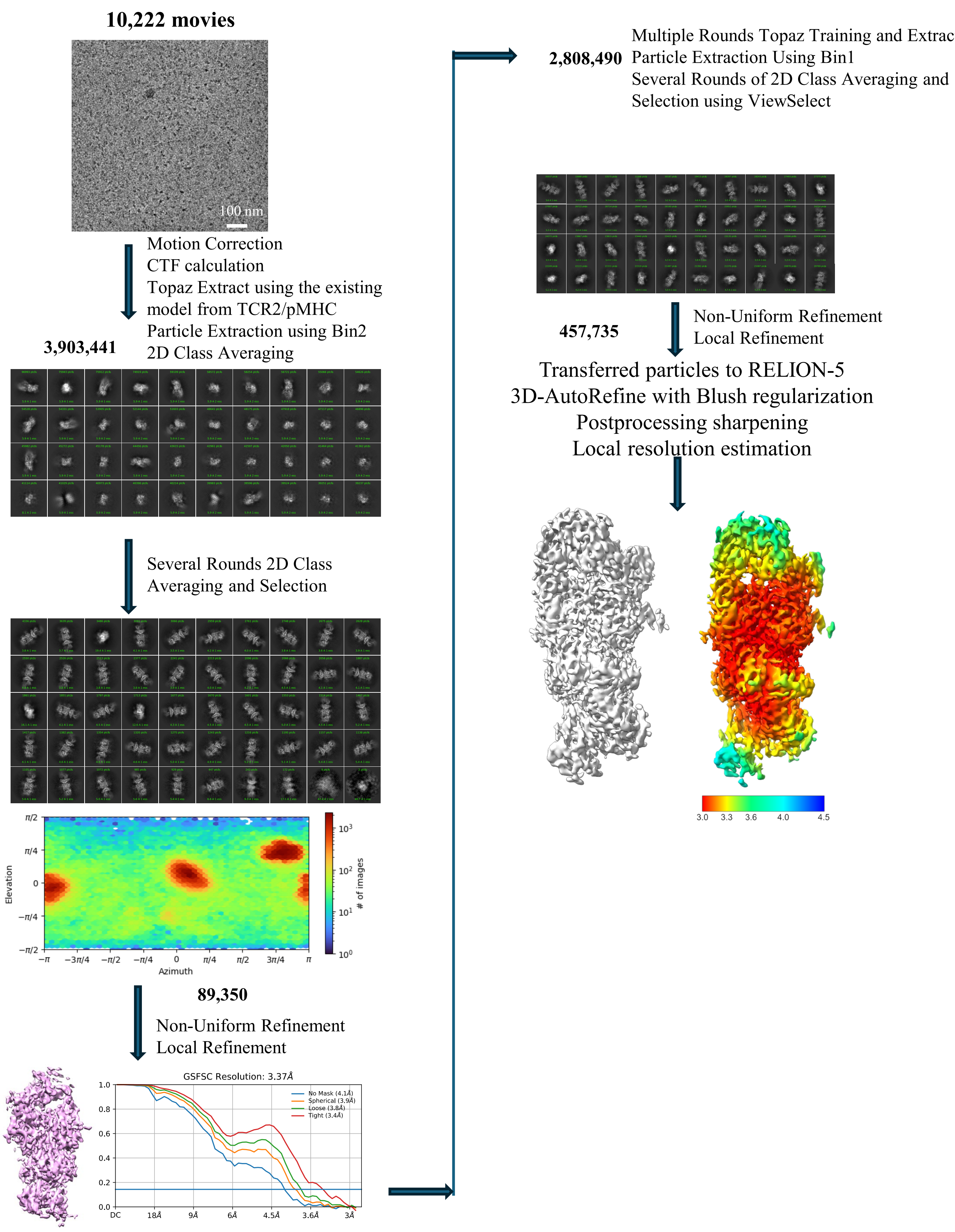

Supplementary Figure 5. Single particle cryo-EM data processing workflow for the TCR_{priv}/pMHC complex.

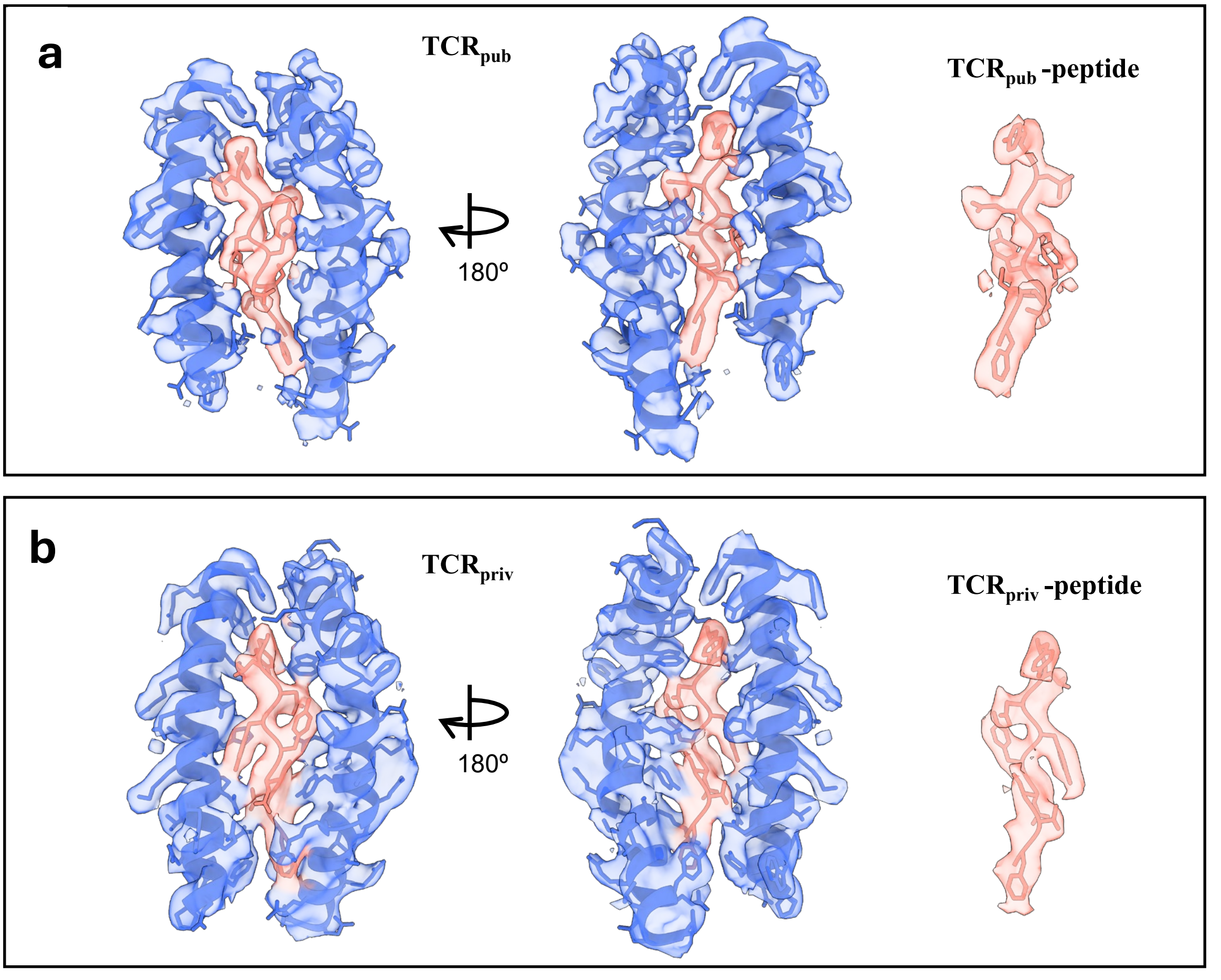

Supplementary Figure 6. Alternative views of Figures 4a and 4d. Structural details of (a) the TCR_{pub}/pMHC and (b) the TCR_{priv}/pMHC complexes, showing the ORF3a(207–215) peptide and MHC interface, with the cryo-EM density map and atomic model superimposed.

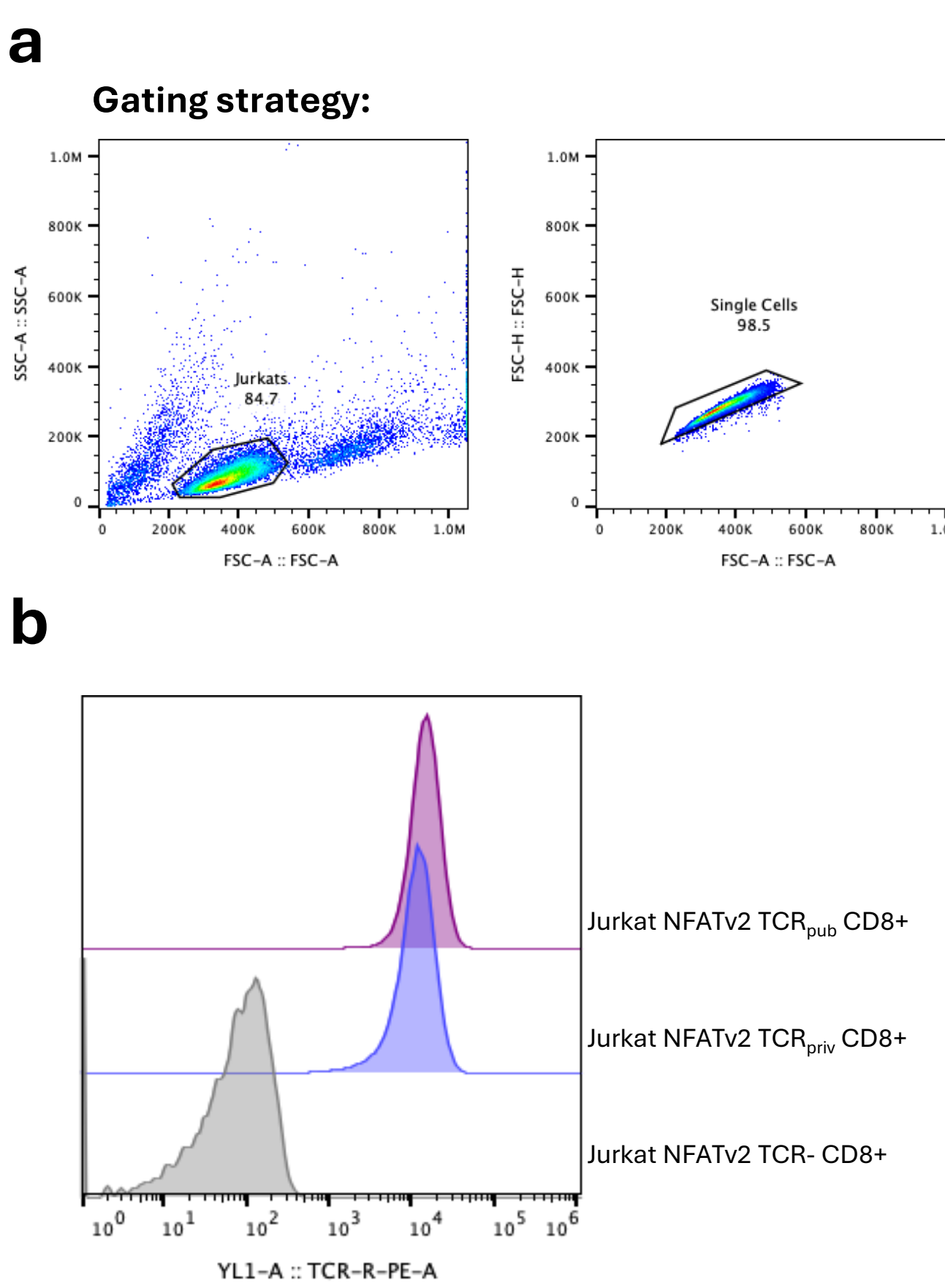

Supplementary Figure 7. TCR expression and validation in Jurkat reporter cells (**a**) Flow cytometry gating strategy for Jurkat CD8^{+} NFAT-luciferase reporter cells. Cells were sequentially gated based on forward and side scatter (FSC/SSC), followed by singlet discrimination and identification of TCR^{+} populations. TCR expression was assessed using a PE-conjugated anti-TCR antibody (clone IP26). (**b**) Histogram showing TCR surface expression, with TCR-positive cells indicated by the gated population and overlay comparison to untransduced control cells (grey).
