# Supplementary material for: Unveiling structural determinants of peptide recognition by public and private SARS-CoV-2-specific T-cell receptors
Source: Nat Commun. 2026 Sep 22;17:11026. doi: 10.1038/s41467-026-77827-z (PMC13687542; doi:10.1038/s41467-026-77827-z)
Supplement: Supplementary file 2 [file 41467_2026_77827_MOESM2_ESM.pdf]

Description of Additional Supplementary Files

Title: Supplementary Data 1

Description: TCR repertoire data from single-cell Smart-seq2 analysis of ORF3a(207–215)-HLA-A*01:01-specific CD8+ T cells isolated from convalescent COVID-19 donors. The file includes paired TCRα/TCRβ clonotype information, TRA and TRB gene usage, CDR3 sequences, donor identifiers, clonotype frequencies, and shared TCR chains used to identify public and private ORF3a-specific TCR clonotypes.

Title: Supplementary Data 2

Description: TCR sequence similarity data from comparison of TCR_{priv} and TCR_{pub} CDR3α and CDR3β sequences with TCR sequences in the OTS COVID-19 TCR database. The file includes paired TCRα/TCRβ sequences with CDR3 similarity scores >0.8 to TCR_{priv} or TCR_{pub}, TRA and TRB V gene usage, CDR3 sequences, and α-β chain pairing information used for the Circos visualizations shown in Fig. 2a-b.
