# Supplementary material for: Unveiling structural determinants of peptide recognition by public and private SARS-CoV-2-specific T-cell receptors
Source: Nat Commun. 2026 Sep 22;17:11026. doi: 10.1038/s41467-026-77827-z (PMC13687542; doi:10.1038/s41467-026-77827-z)
Supplement: Supplementary file 3 [file 41467_2026_77827_MOESM4_ESM.pdf]

nature portfolio

Corresponding author(s): Peijun Zhang

Last updated by author(s): 2026-4-24

Reporting Summary

Nature Portfolio wishes to improve the reproducibility of the work that we publish. This form provides structure for consistency and transparency in reporting. For further information on Nature Portfolio policies, see our Editorial Policies and the Editorial Policy Checklist.

Statistics

For all statistical analyses, confirm that the following items are present in the figure legend, table legend, main text, or Methods section.

n/a	Confirmed	
☐	☒	The exact sample size (n) for each experimental group/condition, given as a discrete number and unit of measurement
☐	☒	A statement on whether measurements were taken from distinct samples or whether the same sample was measured repeatedly
☐	☒	The statistical test(s) used AND whether they are one- or two-sided<br>*Only common tests should be described solely by name; describe more complex techniques in the Methods section.*
☒	☐	A description of all covariates tested
☐	☒	A description of any assumptions or corrections, such as tests of normality and adjustment for multiple comparisons
☐	☒	A full description of the statistical parameters including central tendency (e.g. means) or other basic estimates (e.g. regression coefficient) AND variation (e.g. standard deviation) or associated estimates of uncertainty (e.g. confidence intervals)
☐	☒	For null hypothesis testing, the test statistic (e.g. F, t, r) with confidence intervals, effect sizes, degrees of freedom and P value noted<br>*Give P values as exact values whenever suitable.*
☒	☐	For Bayesian analysis, information on the choice of priors and Markov chain Monte Carlo settings
☒	☐	For hierarchical and complex designs, identification of the appropriate level for tests and full reporting of outcomes
☒	☐	Estimates of effect sizes (e.g. Cohen's d, Pearson's r), indicating how they were calculated

Our web collection on statistics for biologists contains articles on many of the points above.

Software and code

Policy information about availability of computer code

Data collection	ELISpot data were collected with AID ELISpot 7.0; Sequencing data were collected with NextSeq Control Software (NCS) version 4 or MiSeq Control Software Version 2.6.2.1; EPU v3.4
Data analysis	TCR sequences were reconstructed from SmartSeq2 scRNAseq FASTQ files using MiXCR v.3.0.13 and imported into R using immunarch v0.9. Circos plots were generated using circlize package (v0.4.12) , cryosparc V4.2, RELION 5, ChimeraX 1.6.1, WinCoot 0.9.8.7, PHENIX 1.19.2, GraphPad Prism 10, Image J

For manuscripts utilizing custom algorithms or software that are central to the research but not yet described in published literature, software must be made available to editors and reviewers. We strongly encourage code deposition in a community repository (e.g. GitHub). See the Nature Portfolio guidelines for submitting code & software for further information.

Data

Policy information about availability of data

All manuscripts must include a data availability statement. This statement should provide the following information, where applicable:

- Accession codes, unique identifiers, or web links for publicly available datasets
- A description of any restrictions on data availability
- For clinical datasets or third party data, please ensure that the statement adheres to our policy

All data supporting the findings of this study are available within the paper and its Supplementary Information. Cryo-EM maps and atomic models have been

deposited in the Electron Microscopy Data Bank (EMDB) and the RCSB Protein Data Bank (PDB) under the following accession codes: TCRpriv/pMHC map, EMD-54366; model, PDB 9RXM. TCRpub/pMHC map, EMD-54262; model, PDB 9RU5. TCRpub/pMHC dimer map, EMD-54273; model, PDB 9RUP. All datasets are publicly available for download and visualization.

Research involving human participants, their data, or biological material

Policy information about studies with human participants or human data. See also policy information about sex, gender (identity/presentation), and sexual orientation and race, ethnicity and racism.

Reporting on sex and gender	Sex is provided for participants in cohort characteristics table previously published (https://doi.org/10.1038/s41590-021-01084-z0
Reporting on race, ethnicity, or other socially relevant groupings	N/A
Population characteristics	52 individuals were recruited following recovery from COVID-19, including 30 mild cases and 22 severe cases. The characteristics was shown in previous publications ((https://doi.org/10.1038/s41590-020-0782-6), and https://doi.org/10.1038/s41590-021-01084-z0)
Recruitment	Patients were recruited from the John Radcliffe Hospital in Oxford, UK, between March 2020 and April 2021 by identification of patients hospitalised during the SARS-COV-2 pandemic. Patients were recruited into the Sepsis Immunomics study and had samples collected during their convalescence as well as during acute disease. For convalescence, patients were sampled at least 28 days from the start of their symptoms. Written informed consent was obtained from all patients.
Ethics oversight	The samples were collected from patients with confirmed COVID who had consented to participate in Sepsis Immunomic project IRAS 260007. Ethical approval was given by the South Central-Oxford C Research Ethics Committee in England (19/SC/0296).

Note that full information on the approval of the study protocol must also be provided in the manuscript.

Field-specific reporting

Please select the one below that is the best fit for your research. If you are not sure, read the appropriate sections before making your selection.

[x] Life sciences [ ] Behavioural & social sciences [ ] Ecological, evolutionary & environmental sciences

For a reference copy of the document with all sections, see nature.com/documents/nr-reporting-summary-flat.pdf

Life sciences study design

All studies must disclose on these points even when the disclosure is negative.

Sample size	The clinical cohort includes 52 participants recovered from COVID-19 samples (30 mild and 22 severe). This was a follow-up for a published study (Peng et al, NI, https://doi.org/10.1038/s41590-020-0782-6). Samples sizes were based on maximal available samples sets where detailed clinical and serological data were also available, and aligned well with our previously published data in other settings, including influenza and HIV (Lee et al, JCI 2008; Zhang et al, NC 2013; Zhao et al, AJCCM 2012)
Data exclusions	Following the first steps of cryo-EM data processing, a small number of the acquired cryo-EM movies were discarded owing to poor ice quality, excessive movement or defocus. Otherwise no data were excluded.
Replication	All functional assays were carried out at least 3 times. All attempts to replicate were successful
Randomization	We were not carrying out randomized trials. This is a structure-function analysis. Cryo-EM images were collected automatically in an unbiased manner.
Blinding	This is a structure-function analysis using cryo-EM and functional analysis involving biochemical and cell culture-based studies. Cryo-EM images were acquired automatically. Consequently, blinding is not relevant to this study.

Reporting for specific materials, systems and methods

We require information from authors about some types of materials, experimental systems and methods used in many studies. Here, indicate whether each material, system or method listed is relevant to your study. If you are not sure if a list item applies to your research, read the appropriate section before selecting a response.

Materials & experimental systems

n/a	Involved in the study
☐	☒ Antibodies
☐	☒ Eukaryotic cell lines
☒	☐ Palaeontology and archaeology
☒	☐ Animals and other organisms
☒	☐ Clinical data
☒	☐ Dual use research of concern
☒	☐ Plants

Methods

n/a	Involved in the study
☒	☐ ChIP-seq
☐	☒ Flow cytometry
☒	☐ MRI-based neuroimaging

Antibodies

Antibodies used

Antibodies used for flow cytometry

Marker	Fuorophore	Supplier	Cat number	Clonotype	Lot Number	Dilution
CD14	BV510	BioLegend	367124	63D3	B280762	1:50
CD16	BV510	BioLegend	302048	3G8	B289732	1:50
CD19	BV510	BioLegend	302242	HIB19	B281769	1:50
CD8	PerCP-Cy5.5	BD Bioscience	565310	SK1	9312532	1:33
CD3	FITC	BD Bioscience	345763	SK7	0342661	1:25

rabbit anti-ANP32B (Abcam, ab200836); rabbit anti-influenza A virus PB2 (Genetex, GTX125925); rabbit anti-Vinculin (Abcam, ab129002); goat anti-rabbit antibody conjugated to horseradish peroxidase (Genetex, GTX213110-01)

Validation

All antibodies used in this study are commercially available. Antibodies used in a specific species or application have been appropriately validated by manufacturers for that application and this information is provided on their website and product information datasheets. All antibodies described here have been further optimized for an appropriate concentration by testing several dilutions.
CD14 https://www.biolegend.com/en-us/products/brilliant-violet-510-anti-human-cd14-antibody-14983
CD16 https://www.biolegend.com/en-us/products/brilliant-violet-510-anti-human-cd16-antibody-8003
CD19 https://www.biolegend.com/en-us/products/brilliant-violet-510-anti-human-cd19-antibody-8004
CD3 https://www.bdbiosciences.com/sg/reagents/clinical/reagents/single-antibodies/cd3-fitc-sk7-also-known-as-leu-4/p/345763
CD8 https://www.bdbiosciences.com/sg/reagents/research/antibodies-buffers/immunology-reagents/anti-non-human-primate-antibodies/cell-surface-antigens/percp-cy55-mouse-anti-human-cd8-sk1/p/565310
https://www.abcam.com/products/primary-antibodies/phapi2--april-antibody-epr14588-ab200836
https://www.genetex.com/Product/Detail/Influenza-A-virus-PB2-protein-antibody/GTX125925
https://www.abcam.com/products/primary-antibodies/vinculin-antibody-epr8185-ab129002
https://www.genetex.com/Product/Detail/Goat-Anti-Rabbit-IgG-antibody-HRP/GTX213110-01

Eukaryotic cell lines

Policy information about cell lines and Sex and Gender in Research

Cell line source(s)

EBV-transformed B cell lines were established in the lab. Human embryonic kidney 293T cells (HEK-293T), Jurkat CD8+ NFAT-luciferase reporter cells, baby hamster kidney cells stably expressing the T7 polymerase (BSR-T7/5 cells), and Spodoptera frugiperda Sf9 cells were obtained from established laboratory stocks and maintained under standard culture conditions. Cell lines were routinely monitored for expected morphology and growth characteristics.

Authentication

Authentication was not performed for this study.

Mycoplasma contamination

Mycoplasma testing revealed no contamination.

Commonly misidentified lines
(See ICLAC register)

No commonly misidentified cell lines were used.

Plants

Seed stocks: N/A

Novel plant genotypes: N/A

Authentication: N/A

Flow Cytometry

Plots

Confirm that:

- [x] The axis labels state the marker and fluorochrome used (e.g. CD4-FITC).
- [x] The axis scales are clearly visible. Include numbers along axes only for bottom left plot of group (a 'group' is an analysis of identical markers).
- [x] All plots are contour plots with outliers or pseudocolor plots.
- [x] A numerical value for number of cells or percentage (with statistics) is provided.

Methodology

Sample preparation: Cryopreserved PBMCs were thawed and rested overnight in R10 at 37 °C. On the second day, PBMCs were stained with PE-conjugated HLA-A*01:01 ORF3a(207–215) pentamer (ProImmune, Oxford, UK). Live/Dead Fixable Aqua dye (Invitrogen) was used to exclude non-viable cells from the analysis. Subsequently, cells were washed and stained with the following surface antibodies: CD3-FITC (BD Biosciences), CD8-PerCP-Cy5.5, CD14-BV510, CD19-BV510 and CD16-BV510 (BioLegend), as well as a PE-conjugated anti-TCR antibody (clone IP26). After the final wash, cells were resuspended in 500 µl of PBS containing 2 mM EDTA and 0.5% BSA (Sigma-Aldrich) and kept in the dark at 4 °C until flow cytometric acquisition. After exclusion of non-viable/CD19+/CD14+/CD16+ cells, CD3+ CD8+ pentamer+ T cells were identified and sorted directly into 96-well PCR plates containing cell lysis buffer using a BD Fusion 1 or BD FACS Aria III (BD Biosciences). T cell activation was additionally assessed using an NFAT-luciferase reporter assay in Jurkat CD8+ cells transduced with TCR constructs.

Instrument: Samples were sorted using BD Fusion 1 or BD FACS Aria III (BD Biosciences)

Software: Data were collected using FACS DIVA (v9.0.1)

Cell population abundance: All sorted samples were checked for after-sorting purity (>99%)

Gating strategy: For all the experiments, cells were first gated on single Lymphocytes by a forward side scatter gate. On sorting for RNASeq, after excluding dead cells, CD14+, CD19+, and CD16+ cells, HLA-A1-ORF3a 207-215 specific T cells were identified as CD3 +CD8+Pentamer+, with TCR expression assessed using a PE-conjugated anti-TCR antibody (clone IP26)

- [x] Tick this box to confirm that a figure exemplifying the gating strategy is provided in the Supplementary Information.
