# Supplementary material for: Unveiling structural determinants of peptide recognition by public and private SARS-CoV-2-specific T-cell receptors
Source: Nat Commun. 2026 Sep 22;17:11026. doi: 10.1038/s41467-026-77827-z (PMC13687542; doi:10.1038/s41467-026-77827-z)
Supplement: Supplementary file 4 [file 41467_2026_77827_MOESM5_ESM.pdf]

nature portfolio

Peer Review File

Unveiling structural determinants of peptide recognition by public and private SARS-CoV-2-specific T-cell receptors

Corresponding Author: Professor Peijun Zhang

This manuscript has been previously reviewed at another journal. This document only contains information relating to versions considered at Nature Communications.

This file contains all reviewer reports in order by version, followed by all author rebuttals in order by version.

Version 0:

Reviewer comments:

Reviewer #1

(Remarks to the Author)
The authors have addressed the previous comments and questions, repeated the SPR data that is now reaching saturation, and modified the text accordingly.

Reviewer #1 – previous point 1 (suggestion)

The title is better, I would suggest including SARS-CoV-2 in the title.
The argument that TCRpMHC structures can be solved by cryoEM in their soluble unmodified state is acknowledge, however other proteins<100kDa have been solved by cryoEM and this does not represent a technical novelty even if it will indeed be a new way of solving structure of TCRpMHC.

Reviewer #1 – previous point 13 (comment)

Regarding the need of cryoEM for the TCR with TRBV5-1, TRAV3, and TRAV25 is not supported by the current structure available for TRBV5-1+ and TRAV3+ TCRs solved by x-ray crystallography. Also from experience cryoEM is not a silver bullet that will help wolved all the TCRpMHC complexes unfortunately.

COMMENTS
Line 76-78 and 84-86 “Most TCR/pMHC complexes have been solved by X-ray crystallography, a method that favours high-affinity or engineered receptors and relies on extensive recombinant protein production.” And yet most of the structures solved are from wt unmodified TCR sequence, so I think this section and the following lines need to be altered to not suggest that mainly high-affinity engineered TCR structures have been solved. The average of Kd from structure solved is 40uM that is consider moderate affinity for TCR, doi.org/10.3390/ijms22010068.

Line 95 “allele” should be replaced by allomorph as allele do not present peptide.

Line 108. The TCRs studies here have a Kd of 4 and 8 uM and so calling them “low-affinity” is not in line with the field, and as the study report only the cryoEM structure of those 2 TCRs it is no proven that cryoEM can be suitable to solved the structure of low-affinity TCR and this would actually be unlikely.

Looking at the supp excel table with the TCR sequences, there is only 1 public TCR shared between 2 individuals (37 and 59), therefore the following corrections are required:
-in the table the alpha chain is classified as private and this should be public
C-COV19-037 CAVRVVTSGGSYIPTF TRAV3 TRAJ6 CASSLAGDLGTEAFF TRBV5-1 TRBJ1-1 private public
-line 113 title should be “public TCR” or “public TCR chains” or “bias repertoire and public TCR”
-line 126 “only a subset of these constituted public TCR clonotypes (shared complete αβ TCRs).” As there is only 1 public

TCR it should be all single and not plural “only a subset of these constituted public TCR clonotype (shared complete αβ TCR).”
-line 138 “higher frequency (17.5%)” in one donor? In multiple donors? This is unclear

Fig 2e, the Kd are reported in mM (millimolar) on the figure and should be micromolar instead.

Line 174. See my comment above about considering <10uM of Kd a moderate affinity is not in line with the field.

Fig4. The label on the CDR loops should be CDR3 and not CDR 3. Also having the same colour for different TCRs does not make it easy to compare the structures.

Line 241. In the structure analysis of the docking mode something that is perhaps not exploited from the data is the fact that despite the TRBV5-1 bias this TCR segment does not seems to contribute a lot to the overall TCR interaction and therefore might explained the different interaction seen between public and private TCR that have different TRAV.

Reviewer #3

(Remarks to the Author)
I was asked to provide an evaluation of whether the concerns of reviewer 2 from the previous round of peer reviews have been addressed by the revisions provided by the authors, that I outline below:

While I appreciate the authors' efforts to revise the work, I believe that the suggestions/concerns raised by reviewer 2 in comments #3 and #6 are not sufficiently addressed by the revised manuscript.

Specifically, the evaluation of interactions with peptide mimics suggested by the reviewer are indeed feasible and would glean important insights into the antigen recognition mechanism, which is the focus of this study and in my opinion remains incompletely described. Therefore, I disagree with the author's response about the physiological relevance of the modified peptides.

Moreover, I have concerns about the quality of the first cryoEM structure in Fig. 3a, which is completely lacking density for alpha3/b2m which is typically a highly stable part of an MHC-I complex.

Overall, while I do find the work very interesting and relevant for the T cell and structural immunology fields, there remain important limitations that should be addressed prior to publication.

Version 1:

Reviewer comments:

Reviewer #1

(Remarks to the Author)
Thank you to the authors for their careful revision and for thoroughly addressing my previous comments and questions. I have no further remarks. One minor suggestion would be to clarify the meaning of the dotted line in Figure 5j within the figure legend.

Reviewer #3

(Remarks to the Author)
I appreciate the reviewer's response to my first comment supported by their new Alanine scan data. Regarding my second comment on the quality of their cryoEM map and model, I agree that given that their analysis focuses on the TCR/peptide/alpha1/2 interface the missing structure of b2m./alpha3 in their deposited model is not critical. However, for reproducibility purposes, the authors should provide explicit details on the changes they introduced to their original cryoEM map to derive a map which completely omits the corresponding alpha3/beta2m densities and the contour levels set for the respective maps.

Open Access This Peer Review File is licensed under a Creative Commons Attribution 4.0 International License, which permits use, sharing, adaptation, distribution and reproduction in any medium or format, as long as you give appropriate credit to the original author(s) and the source, provide a link to the Creative Commons license, and indicate if changes were made.
In cases where reviewers are anonymous, credit should be given to 'Anonymous Referee' and the source.
The images or other third party material in this Peer Review File are included in the article's Creative Commons license, unless indicated otherwise in a credit line to the material. If material is not included in the article's Creative Commons license and your intended use is not permitted by statutory regulation or exceeds the permitted use, you will need to obtain permission directly from the copyright holder.
To view a copy of this license, visit https://creativecommons.org/licenses/by/4.0/

Point-by-point responses to reviewers' comments

Reviewer #1 (Remarks to the Author):

1- The authors have addressed the previous comments and questions, repeated the SPR data that is now reaching saturation, and modified the text accordingly.

We thank the reviewer for this positive assessment.

2- Reviewer #1 – previous point 1 (suggestion)
The title is better, I would suggest including SARS-CoV-2 in the title.

We thank the reviewer for this helpful suggestion. We have therefore revised the title to: **"Uncovering structural determinants of peptide recognition by public and private SARS-CoV-2-specific T-cell receptors"**

3- The argument that TCRpMHC structures can be solved by cryoEM in their soluble unmodified state is acknowledge, however other proteins<100kDa have been solved by cryoEM and this does not represent a technical novelty even if it will indeed be a new way of solving structure of TCRpMHC.

We agree that determining structures of macromolecular assemblies below 100 kDa by cryo-EM is not, in itself, unprecedented, and we did not intend to suggest that this size range constitutes a general technical breakthrough. We have therefore revised the manuscript to clarify this point and avoid overstatement. Our intention is to demonstrate that cryo-EM can be successfully applied to wild-type soluble TCR/pMHC complexes, a class of samples that has not been commonly studied by this method. We now present this more carefully as a complementary approach to crystallography for structural analysis of TCR/pMHC complexes. The Introduction and Discussion have been revised accordingly.

4- Reviewer #1 – previous point 13 (comment)
Regarding the need of cryoEM for the TCR with TRBV5-1, TRAV3, and TRAV25 is not supported by the current structure available for TRBV5-1+ and TRAV3+ TCRs solved by x-ray crystallography. Also from experience cryoEM is not a silver bullet that will help wolved all the TCRpMHC complexes unfortunately.

We thank the reviewer for this thoughtful comment and agree with both points raised. We have therefore revised the text to remove any implication that these specific V genes intrinsically require cryoEM for structural study. Indeed, cryoEM is not a universal solution; rather, we aim to present it as a complementary structural approach that can be particularly valuable when crystallization is challenging or when preservation of the native soluble complex is advantageous. We have revised the relevant text in the Introduction and Discussion to make this point explicit and to avoid overgeneralization.

COMMENTS
5- Line 76-78 and 84-86 "Most TCR/pMHC complexes have been solved by X-ray crystallography, a method that favours high-affinity or engineered receptors and relies on extensive recombinant protein production." And yet most of the structures solved are from wt unmodified TCR sequence, so I think this section and the following lines need to be altered to not suggest that mainly high-affinity engineered TCR structures have been solved. The average

of Kd from structure solved is 40uM that is consider moderate affinity for TCR, doi.org/10.3390/ijms22010068.

We thank the reviewer for this important correction. We have revised this section to state more accurately that X-ray crystallography has provided the majority of currently available TCR/pMHC structures, including many involving wild-type receptors, while structural coverage of the broader repertoire remains incomplete.

We have also revised the affinity-related discussion throughout the manuscript to better reflect the typical affinity range represented in the current structural database, as the reviewer noted.

6- Line 95 “allele” should be replaced by allomorph as allele do not present peptide.

“Allele” has been replaced with **“allomorph”**.

7- Line 108. The TCRs studies here have a Kd of 4 and 8 uM and so calling them “low-affinity” is not in line with the field, and as the study report only the cryoEM structure of those 2 TCRs it is no proven that cryoEM can be suitable to solved the structure of low-affinity TCR and this would actually be unlikely.

We agree with the reviewer and have revised the manuscript accordingly to describe these complexes consistently as micromolar-affinity throughout. Our conclusions are now limited to what is directly supported by the present data: namely, that cryoEM can resolve the structures of these two wild-type, micromolar-affinity TCR/pMHC complexes and provide interpretable information about their recognition interfaces.

8- Looking at the supp excel table with the TCR sequences, there is only 1 public TCR shared between 2 individuals (37 and 59), therefore the following corrections are required: in the table the alpha chain is classified as private and this should be public
C-COV19-037 CAVRVVTSGGSYIPTF TRAV3 TRAJ6 CASSLAGDLGTEAFF TRBV5-1 TRBJ1-1 private public

We thank the reviewer for this careful observation. We have checked the supplementary TCR table and confirmed that the clonotype shared between donors C-COV19-037 and C-COV19-059 is a public paired TCR. Accordingly, we have corrected the annotation in Supplementary Table 2, updating the alpha chain classification from “private” to “public” for this entry.

9- line 113 title should be “public TCR” or “public TCR chains” or “bias repertoire and public TCR”

We thank the reviewer for this suggestion. We have revised the subheading to:

“Dominant SARS-CoV-2 ORF3a(207–215)-A*01:01-specific T cells show biased TCR usage and include a public TCR clonotype”

10- line 126 “only a subset of these constituted public TCR clonotypes (shared complete αβ TCRs).” As there is only 1 public TCR it should be all single and not plural “only a subset of these constituted public TCR clonotype (shared complete αβ TCR).”

We thank the reviewer. This has been corrected to the singular throughout the manuscript when referring specifically to the dataset analysed here.

11- line 138 "higher frequency (17.5%)" in one donor? In multiple donors? This is unclear

We thank the reviewer for highlighting this ambiguity. The frequency referred to the proportion of this clonotype within the antigen-specific repertoire of a single donor, not across multiple donors. We have revised the text to state this explicitly and now specify the donor identity in the sentence and/or figure legend to avoid any confusion.

12- Fig 2e, the Kd are reported in mM (millimolar) on the figure and should be micromolar instead.
We thank the reviewer for spotting this labeling error. This has been corrected in the revised figure.

13- Line 174. See my comment above about considering <10uM of Kd a moderate affinity is not in line with the field.

We thank the reviewer and agree. Please see the responses to point 5 and 7.

14- Fig4. The label on the CDR loops should be CDR3a and not CDRa3. Also having the same colour for different TCRs does not make it easy to compare the structures.

We thank the reviewer for these helpful suggestions. We have corrected the CDR loop labels throughout the manuscript, figures, and figure legends to follow standard nomenclature.

Regarding the colour scheme, we have increased the visibility of the CDR loop labels and refined the presentation to better highlight key structural features. Although we considered using additional colour schemes to distinguish the different TCRs, we chose to retain a consistent palette to avoid overcomplicating the figures, as several structural elements are already colour-coded. In addition, Figure 5 provides a more detailed comparison, including superposition of the complexes as well as CDR- and residue-level colouring, which allows clearer distinction between them.

15- Line 241. In the structure analysis of the docking mode something that is perhaps not exploited from the data is the fact that despite the TRBV5-1 bias this TCR segment does not seems to contribute a lot to the overall TCR interaction and therefore might explained the different interaction seen between public and private TCR that have different TRAV.

We thank the reviewer for this insightful observation and agree that this is an important point. We have revisited the structural analysis to clarify this aspect. While both TCRs share the same TRBV5-1, differences in the CDR3β sequences (AGDL in TCR_{pub} versus WTGV in TCR_{priv}) contribute to distinct interaction footprints at the interface, including both peptide and MHC contacts. At the same time, differences in α-chain usage further shape the docking geometry through the CDR loops. We have therefore revised the text to emphasize that the divergent recognition modes arise from the combined contributions of both chains, rather than from α-chain usage alone.

Reviewer #3 (Remarks to the Author):

I was asked to provide an evaluation of whether the concerns of reviewer 2 from the previous round of peer reviews have been addressed by the revisions provided by the authors, that I outline below:

16- While I appreciate the authors' efforts to revise the work, I believe that the suggestions/concerns raised by reviewer 2 in comments #3 and #6 are not sufficiently addressed by the revised manuscript.

Specifically, the evaluation of interactions with peptide mimics suggested by the reviewer are indeed feasible and would glean important insights into the antigen recognition mechanism, which is the focus of this study and in my opinion remains incompletely described. Therefore, I disagree with the author's response about the physiological relevance of the modified peptides.

We have now performed additional peptide alanine-scanning experiments. These data directly assess the contributions of peptide side chains that are differentially engaged by TCR_{pub} and TCR_{priv}, and they provide functional support for the distinct recognition modes inferred from the structures. Specifically, TCR_{pub} is more sensitive to alanine substitutions at the key D4 position than TCR_{priv}, consistent with the structural data. The results are included in the revised manuscript (new Fig. 5j, Supplementary Fig7).

17- Moreover, I have concerns about the quality of the first cryoEM structure in Fig. 3a, which is completely lacking density for alpha3/b2m which is typically a highly stable part of an MHC-I complex.

We thank the reviewer for this observation. It is indeed notable that the MHC α3 domain and β2-microglobulin in the TCR_{pub}/pMHC complex are comparatively poorly resolved, despite this region typically being well ordered in MHC-I structures. In contrast, this region is well resolved in the TCR_{priv}/pMHC complex, suggesting differences in structural stability or conformational heterogeneity between the two complexes.

Our analysis focused on the TCR/peptide/MHC α1/α2 interface together with the underlying MHC β-sheet platform, and for both complexes we obtained maps of sufficient quality to support confident model building of the TCR, peptide, and MHC α1/α2 domains that form the antigen-recognition interface. Importantly, our structural conclusions are based on this well-resolved region and do not depend on the membrane-proximal α3/β2m portion. We have revised the manuscript accordingly to clarify the basis of our structural interpretation.

18- Overall, while I do find the work very interesting and relevant for the T cell and structural immunology fields, there remain important limitations that should be addressed prior to publication.

We appreciate the reviewer’s comment and have revised the manuscript to address the limitations.

Point-by-point responses to reviewers' comments

Reviewer #1 (Remarks to the Author):

Thank you to the authors for their careful revision and for thoroughly addressing my previous comments and questions. I have no further remarks. One minor suggestion would be to clarify the meaning of the dotted line in Figure 5j within the figure legend.

We thank the reviewer for the positive assessment of our revised manuscript and for this helpful suggestion. We have revised the legend of Figure 5j to explicitly explain the meaning of the dotted line.

Reviewer #3 (Remarks to the Author):

I appreciate the reviewer's response to my first comment supported by their new Alanine scan data. Regarding my second comment on the quality of their cryoEM map and model, I agree that given that their analysis focuses on the TCR/peptide/alpha1/2 interface the missing structure of b2m./alpha3 in their deposited model is not critical. However, for reproducibility purposes, the authors should provide explicit details on the changes they introduced to their original cryoEM map to derive a map which completely omits the corresponding alpha3/beta2m densities and the contour levels set for the respective maps.

We thank the reviewer for the positive assessment and helpful comment. We have expanded the cryo-EM single-particle analysis section in the Methods to provide additional details on the focused classification/refinement procedure used for the TCR_{pub}/pMHC map. In addition, we have included the contour levels used for visualization of the cryo-EM maps in the relevant figure legends.
